# A theoretical and computational framework for studying creep crack growth

**DOI:** 10.1007/s10704-017-0230-2

**Published:** 2017-08-07

**Authors:** Elsiddig Elmukashfi, Alan C. F. Cocks

**Affiliations:** Department of Engineering Science, University of Oxford, Park Road, OX1 3PJ Oxford, UK

**Keywords:** Creep, Crack, C*-integral, Damage zone model, Traction-separation rate law (TSRL), Double cantilever beam (DCB), Dimensionless analysis

## Abstract

In this study, crack growth under steady state creep conditions is analysed. A theoretical framework is introduced in which the constitutive behaviour of the bulk material is described by power-law creep. A new class of damage zone models is proposed to model the fracture process ahead of a crack tip, such that the constitutive relation is described by a traction-separation rate law. In particular, simple critical displacement, empirical Kachanov type damage and micromechanical based interface models are used. Using the path independency property of the $C^*$-integral and dimensional analysis, analytical models are developed for pure mode-I steady-state crack growth in a double cantilever beam specimen (DCB) subjected to constant pure bending moment. A computational framework is then implemented using the Finite Element method. The analytical models are calibrated against detailed Finite Element models. The theoretical framework gives the fundamental form of the model and only a single quantity $\hat{C}_k$ needs to be determined from the Finite Element analysis in terms of a dimensionless quantity $\phi _0$, which is the ratio of geometric and material length scales. Further, the validity of the framework is examined by investigating the crack growth response in the limits of small and large $\phi _0$, for which analytical expression can be obtained. We also demonstrate how parameters within the models can be obtained from creep deformation, creep rupture and crack growth experiments.

## Introduction

At elevated temperature, creep crack growth (CCG) is one of the most common failure mechanisms in many engineering applications, e.g. structural components, similar and dissimilar metal welds etc. This problem has received much attention over the last forty years due to the importance in designing structures with high integrity and safety. Hence, developing analytical models for steady-state crack growth which can be calibrated against detailed Finite Element models is of great interest. Further, an assessment of the effect of different material parameters and damage development processes on the crack growth behaviour can be provided using such models.

Studying creep crack growth has a long history in the literature. A major feature of these studies is the development of a parameter that characterizes the crack tip fields as well as crack propagation. Under steady state creep conditions, the so called $C^*$-integral (Landes and Begley 1976; Nikbin et al. 1976; Ohji et al. 1976) (i.e. the creep *J*-integral, Rice 1978) can be used to characterize the crack tip fields and creep crack growth. It provides descriptions of the strain-rate and stress singularities at the crack tip and a correlation of experimental crack growth rate data (Taira et al. 1979; Riedel and Rice 1980). Moreover, the $C^*$-integral is path independent for contours in which the material properties only vary in the direction perpendicular to the direction of crack growth within the family of contours considered. Riedel and Rice (1980) studied the transition from short-time elastic to long-time creep behaviour assuming that primary creep is negligible (small-scale creep conditions). They introduced a parameter *C*(*t*) that describes the strain, strain-rate and stress fields within a creep zone that forms about the crack tip. Their analysis also provides a characteristic time for the transition to the steady state stress field (i.e. the time for *C*(*t*) to equal $C^*$). Later, Ehlers and Riedel (1981) proposed a relation between *C*(*t*) and $C^*$. Saxena (1986) proposed a new parameter $C_{\mathrm {t}}$ which can be measured easily in comparison with *C*(*t*). Bassani et al. (1988) compared these two parameters and concluded that $C_{\mathrm {t}}$ characterizes crack growth rates much better than *C*(*t*). Further, the *C*(*t*) parameter is found to be more suitable for characterizing a stationary crack and $C_{\mathrm {t}}$ is related to a rapidly propagating crack. In the primary creep regime, Riedel (1981) suggested a new parameter $C^{*}_{h}$ as an analogy to the $C^*$-integral. Further, Leung and McDowell (1990) included the primary creep effects in the estimation of the $C_{\mathrm {t}}$ parameter. To this end, the $C^*$, *C*(*t*), $C_{\mathrm {t}}$ and $C^{*}_{h}$ parameters are generally accepted and widely used in studying creep crack growth.

Under creep conditions, cracks in polycrystalline materials advance as a result of the growth of damage ahead of the crack tip (generally in the form of discrete voids or microcracks, which form primarily at grain boundaries). In the vicinity of a macroscopic primary crack tip, secondary micro-cracks are formed as a result of intensive void growth and coalescence and/or an accumulation and growth of micro-cracks. These secondary cracks propagate and coalesce creating the new crack surfaces, allowing the primary macroscopic crack to advance along an interface or interconnected grain-boundaries. The growth of damage can influence the constitutive properties of the material and therefore the details of the near crack tip stress and strain-rate fields. Early models of creep crack growth either assumed that the stress (e.g. Riedel 1981; Tvergaard 1984) or strain-rate field (e.g. Cocks and Ashby 1981; Nikbin et al. 1984) is the same as that for the undamaged material and used either empirical or mechanistic damage growth laws to determine the crack growth rate. In the strain based models the critical damage at the crack tip is expressed in terms of a material ductility (strain to failure) which is a function of the local stress state. Extensions of this approach within a finite element framework (employing models in which the constitutive relationships for deformation are not influenced by the presence of damage) have been undertaken by Nikbin et al. (1976, 1984), Yatomi and Nikbin (2014). Studies of the influence of damage on the nature of the crack tip fields and crack growth process where damage influences the deformation response have been undertaken by Riedel (1987) and Bassani and Hawk (1990) for empirical Kachanov (Kachanov 1958; Rabotnov 1969) type continuum damage mechanics models. More recently, the full interaction between deformation and damage development and how this influences the crack growth process has been modelled directly using the finite element method, using both mechanistic and empirical models for the growth of damage (e.g. Onck and van der Giessen 1998; Wen and Shan-Tung 2014).

In each of the above referenced studies damage development and its influence on crack growth is modelled as a continuum process. Another method of modelling crack propagation is through the use of interface cohesive or damage zone models. Interface damage zone models of this type provide a coupling between the local separation rate across an interface and bulk deformation processes, i.e. they introduce a physically meaningful length scale that is related to the dissipative mechanisms responsible for damage development. A damage zone model of this type describes the fracture process in the vicinity of the crack tip as a gradual surface separation process, such that the normal and shear tractions at the interface resist separation and relative sliding. The cohesive/damage zone modelling approach has its origins in the pioneering work of Dugdale (1960) and Barenblatt (1962). The first use of cohesive zone models in a finite element environment was undertaken by Hillerborg et al. (1976). Several models have been proposed in the literature, wherein a variety of materials and applications have been successfully investigated (Camacho and Ortiz 1996; Elmukashfi and Kroon 2014; Hui et al. 1992; Knauss 1993; Needleman 1987, 1990; Rahul-Kumar et al. 1999; Rice and Wang 1989; Tvergaard 1990; Xu and Needleman 1993). Rate-dependent and rate-independent models as well as physically based and phenomenological models have been employed. However, to the authors’ knowledge, apart from the work of Onck and van der Giessen (1998), van der Giessen and Tvergaard (1994), Thouless et al. (1983) and Yu et al. (2012) damage zone type models have not been used to study the development of creep damage and/or creep crack growth.

In this paper, a theoretical and computational framework for creep crack growth is presented in which we assume that all the damage is concentrated in a narrow zone directly ahead of the growing crack tip. The objective is to model crack propagation in materials that exhibit steady state creep behaviour outside of the damage zone and to investigate the effect of different material parameters, forms of damage zone constitutive law and damage development processes on the crack growth behaviour. A theoretical framework is initially introduced in which the constitutive behaviour of the bulk material is described by power-law creep. A new class of damage zone model is proposed to model the fracture process such that the constitutive relation is described by a traction-separation rate law. More specifically, three different models, i.e. a simple critical displacement model, Kachanov type empirical models and a micromechanical based interface model are investigated, which mirror the types of models employed in the continuum models of creep crack growth described above. We follow the recent approach of Wang et al. (2016), who studied cleavage failure in creeping polymers, in which we keep the material descriptions and geometric configurations as simple as possible to explore the relationship between the form of the constitutive model, material parameters and crack growth. With this in mind, we concentrate initially on the behaviour of a double cantilever beam specimen (DCB) of infinite length subjected to a constant pure bending moment, in which $C^*$ remains constant as the crack grows and the crack growth rate eventually achieves a steady-state. We concentrate on the behaviour in the steady state. By invoking the path independence of the $C^*$-integral and choosing contours in the far field and surrounding the damage zone we demonstrate how a simple analytical expression for the crack growth rate can be obtained in terms of $C^*$, damage zone material parameters and a dimensionless scaling parameter that is a function of the ratio of characteristic geometric and material length scales, that can be determined using the finite element method. The theoretical framework is presented in Sect. 2. The analysis of creep crack growth in the double cantilever beam specimen and the finite element implementation of the damage zone model are described in Sect. 3, with the crack growth results for the different interface models presented and discussed in Sect. 4.

## Theoretical framework for creep crack

### Background

Consider a solid containing a stationary crack that is subjected to a constant load. The solid is assumed to exhibit elastic behaviour, together with primary, secondary and tertiary creep. Creep deformation evolves with increasing time and this evolution can be divided into different distinct stages. These stages have been described and evaluated by Bassani and Hawk (1990). Initially, a small-scale creep zone, i.e. small in comparison with the physical characteristic length of the body, is formed in the vicinity of the crack tip. In this stage, the material deforms by primary creep inside the creep zone and remains elastic elsewhere. Following the development of the primary creep zone, a secondary (steady-state) creep zone develops as a smaller region inside the primary creep zone. Thereafter, the primary and secondary creep zones continue to expand at the cost of the elastic and primary zones, respectively. During this process, damage accumulates in the crack tip region, which may lead to crack propagation if a critical condition is met. Hence, crack propagation may take place at different instants during the evolution of the near tip stress and strain-rate fields. The crack propagation scenarios are characterized by the nature of the crack tip fields at these instants: (i) the small-scale creep zone is formed surrounded by the elastic medium, (ii) the primary creep zone is large enough but remains surrounded by the elastic medium, (iii) the secondary creep zone is formed inside the primary creep zone but both zones remain surrounded by the elastic medium, (iv) the secondary creep zone is expanding inside the primary creep zone which dominates, (v) the secondary creep zone dominates.

This study concerns crack propagation in creeping materials under steady state conditions (type v). In this case, the $C^*$-integral can be used to characterize the creep crack growth behaviour. Note also, that as a crack grows in an elastic/creeping material, in the absence of damage, a zone develops ahead of the crack tip in which the stresses are determined by the elastic and creep properties of the material (Hui and Riedel 1981) who’s size is a function of the crack velocity. For steady state behaviour the size of this zone must be small compared to the size of the crack tip damage process zone. Under these conditions, the path independent property can be used to obtain a direct relationship between the far field loading and the fracture process parameters.

In the following sub-sections we describe the constitutive relationships for the bulk continuum response and introduce a number of different models to describe the response ahead of the crack tip within the damage zone. We concentrate on mode I crack growth and only present relationships for the opening mode, although a description of the shear response is also required for the computational studies presented later. We consider the crack growth process in Sect. 3, where we use the path independence of the $C^*$-integral to relate the near crack tip damaging processes to the far field loading. In the theoretical models presented below we concentrate on steady state crack growth, where creep dominates the material response, i.e. we need not consider the elastic response. Similarly we do not need to consider any elastic/reversible contributions to the deformation within the damage zone.

### Creep deformation and characterization of the remote field

**Fig. 1:**
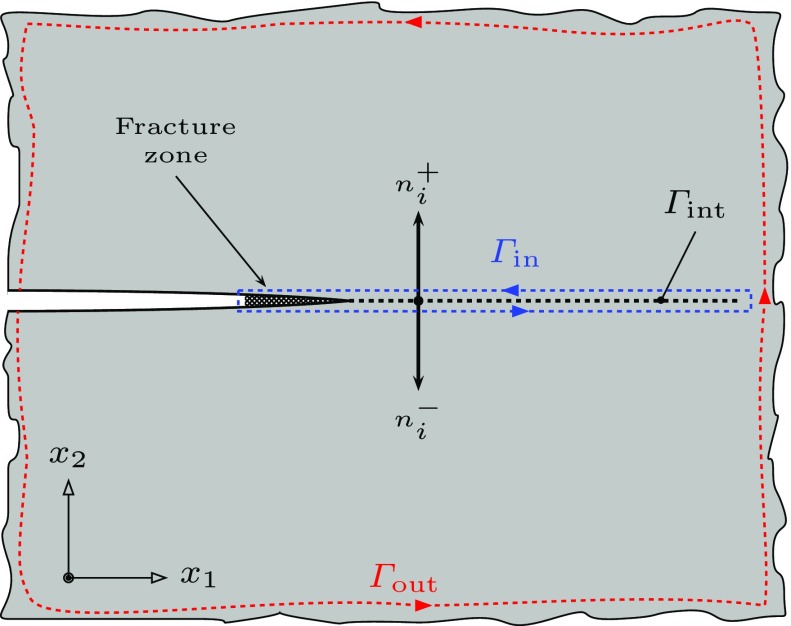
The schematic of interface crack model in creeping solid. The figure illustrates the definition of the interface surface $\varGamma _{\mathrm {int}}$. inner path $\varGamma _{\mathrm {in}}$ and the outer path $\varGamma _{\mathrm {out}}$

Consider a body containing a crack and subjected to a constant far field loading, see Fig. 1. A common Cartesian coordinate system for the reference and deformed configurations $x_i$, $i=1,2,3$, is assumed. The bulk material is assumed to exhibit steady-state creep behaviour and is defined by the constitutive law

1 $$\begin{aligned} \dot{\varepsilon }_{ij} = \dfrac{\partial \phi }{\partial \sigma _{ij}} = \frac{3}{2} \, \dot{\varepsilon }_0 \, \Big (\frac{\sigma _{\mathrm {e}}}{\sigma _0}\Big )^{n} \, \frac{s_{ij}}{\sigma _{\mathrm {e}}}, \end{aligned}$$

where $\sigma _{ij}$ is Cauchy’s stress tensor, $\dot{\varepsilon }_{ij}$ is the strain rate tensor, $s_{ij}=\sigma _{ij}-\frac{1}{3} \sigma _{kk} \delta _{ij}$ is the stress deviator, $\sigma _{\mathrm {e}}=\sqrt{\frac{3}{2} s_{ij} s_{ij}}$ is the von Mises equivalent stress, $\sigma _0$ is a reference stress, $\dot{\varepsilon }_0$ is the strain-rate at the reference stress and *n* is the rate sensitivity parameter. $\phi$ is the stress potential

2 $$\begin{aligned} \phi = \dfrac{1}{n+1} \, \dot{\varepsilon }_{0} \sigma _{0} \left( \dfrac{\sigma _{\mathrm {e}}}{\sigma _{0}}\right) ^{n+1}. \end{aligned}$$

The energy dissipation rate, $\dot{D}$, is given by

3 $$\begin{aligned} \dot{D} = \phi +\psi , \end{aligned}$$

where $\psi$ is the dual rate potential

4 $$\begin{aligned} \psi = \dfrac{n}{n+1} \, \dot{\varepsilon }_{0} \sigma _{0} \left( \dfrac{\dot{\varepsilon }_{\mathrm {e}}}{\dot{\varepsilon }_{0}}\right) ^{\frac{n+1}{n}}, \end{aligned}$$

and $$\dot{\varepsilon }_{\mathrm {e}}=\sqrt{\frac{2}{3} \dot{\varepsilon }_{ij} \dot{\varepsilon }_{ij}}$$.

Crack propagation is assumed to be determined by an interface model such that the propagation takes place along a fictitious interface surface, $\varGamma _{\mathrm {int}}$. As the crack advances separation occurs along the interface to create two surfaces. Hence, a material point along the interface is defined by the two normal vectors $n_{i}^{-}$ and $n_{i}^{+}$, where $n_{i}^{-}=-n_{i}^{+}$, i.e. the initially intact material point splits into two points with unit normals acting opposite to each other and into the material on either side of the interface. The displacement-rate jump across the damage zone surface and the corresponding tractions are defined by the vectors $\delta _{i} = u_{i}^{+}-u_{i}^{-}$, where $u_{i}^{+}$ and $u_{i}^{-}$ are the displacement rates either side of the interface, and $T_{i}^{+}=\sigma _{ij} \, n_{j}^{+}$, $T_{i}^{-}=\sigma _{ij} \, n_{j}^{-}$.

**Fig. 2:**
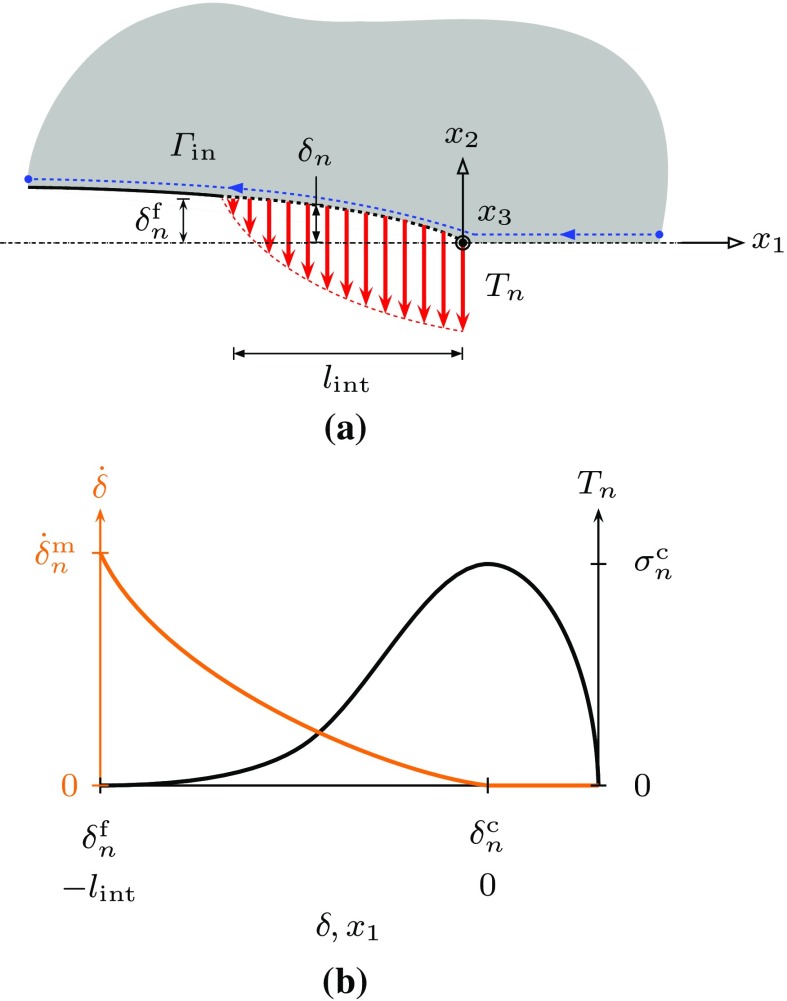
The cohesive zone for pure mode I crack propagation: **a** schematic of the cohesive zone; and **b** the normal traction-separation ($T_{n}-\delta _{n}$) and the separation rate-separation ($\dot{\delta }_{n}-\delta _{n}$) distribution along the cohesive zone. $l_{\mathrm {int}}$ is the length of the cohesive zone, $\delta ^{\mathrm {c}}_{n}$ is the critical displacement, $\delta ^{\mathrm {f}}_{n}$ is the displacement at failure, and $\sigma ^{\mathrm {c}}_{n}$ is the cohesive strength

In order to analyse the problem the $C^*$-integral is determined on the inner and the outer contours in Fig. 1. The $C^*$-integral is defined as

5 $$\begin{aligned} C^* = \int \limits _{\varGamma } W \cdot \mathrm {d}x_2 - T_i \, \frac{\partial \dot{u}_i}{\partial x_1} \cdot \mathrm {d}s, \end{aligned}$$

where $\varGamma$ is an arbitrary contour around the tip of the crack with unit outward normal $n_{i}$, $T_{i} = \sigma _{ij} \, n_{j}$ is the traction on $\mathrm {d}s$ and $\dot{u}_{i}$ is the displacement rate. The $C^*$-integral in the outer path, $C^{*}_{\mathrm {out}}$, is determined by the far field loading. We consider the situation where a damage zone extends along the $x_{1}$ axis directly ahead of the crack tip, see Fig. 2. The first term of Eq. (5) then vanishes, since $\mathrm {d}x_{2} =0$. The contribution of the damage region to the $C^*$-integral along the inner path, $C^{*}_{\mathrm {in}}$, is then evaluated as

6 $$\begin{aligned} C_{\text {in}}^{*}&= -\int \limits _{\varGamma _{\mathrm {in}}}{T_{i}} \frac{\partial \dot{u}_{i}}{\partial x_{1}} \cdot \mathrm {d}s \nonumber \\&= -\int \limits _{0}^{L} \sigma _{ij} n_{i}^{-} \frac{\partial \dot{u}_{i}^{-}}{\partial x_{2}} \cdot \mathrm {d}x_{1}+ \int \limits _{L}^{0} \sigma _{ij} n_{i}^{+} \frac{\partial \dot{u}_{i}^{+}}{\partial x_{1}} \cdot \mathrm {d}x_{1} \nonumber \\&= -\int \limits _{0}^{L} \sigma _{ij} n_{i}^{+} \frac{\partial \dot{\delta }}{\partial x_{1}} \cdot \mathrm {d}x_{1} \nonumber \\&= \int \limits _{0}^{\dot{\delta }_{i}^{m}}{\sigma _{ij}} n_{i}^{+} \cdot \mathrm {d}\dot{\delta }_{i} = \int \limits _{0}^{\dot{\delta }_{i}^{m}} T_{i}^{+}\cdot \mathrm {d}\dot{\delta }_{i}, \end{aligned}$$

where $\dot{\delta }^{\mathrm {m}}_{n}$ is the opening rate at the tip of the crack. In order to simplify the relationships used in subsequent analysis we omit the superscript “$+$” from the traction. Using the path-independence property of $C^*$ (ie, $C^{*}_{\mathrm {out}}=C^{*}_{\mathrm {in}}$) provides a relationship between the far field loading and the behaviour within the damage zone. In order to complete the analysis we need a constitutive relationship for the damage zone that relates $\dot{\delta }_{i}$ to $T_{i}$. In this paper we concentrate on mode I loading and therefore only need to consider the components of traction and displacement-rate normal to the damage zone. Then

7 $$\begin{aligned} C^{*}_{\mathrm {in}} = \int \limits ^{\dot{\delta }^{\mathrm {m}}_{n}}_{0} T_n \cdot \mathrm {d}\dot{\delta }_n. \end{aligned}$$

In the following sub-section we present a number of different constitutive relationships for the damage zone response.

### Damage zone models for creep crack growth

In this section, we present models for the damage zone. We limit our description to mode I loading. More general relationship for mode II and mixed mode loading are described elsewhere (Elmukashfi and Cocks 2017). For each of the models here we assume that the relationship between $\dot{\delta }_{n}$ and $T_{n}$ can be expressed in the form of a power-law. Further, these models are capable of predicting similar damaging processes and crack growth behaviour through the appropriate selection of material parameters, see “Appendix B”.

#### Simple critical displacement model

The simplest form of traction-separation rate law is defined by a direct power-law relationship between the normal traction $T_{n}$ and the separation rate $\dot{\delta }_{n}$. Further, damage does not influence the opening rate and failure is achieved when $\delta _{n}$ achieves a critical separation $\delta ^{\mathrm {f}}_{n}$. Thus, the constitutive model takes the following form

8 $$\begin{aligned} \dot{\delta }_{n} = \dot{\delta }_{0} \left( \dfrac{T_{n}}{T_{0}}\right) ^{m}, \end{aligned}$$

where $T_{0}$ is a reference traction (equivalent to of Eq. (1), $\dot{\delta }_{0}$ is the separation rate at this traction and *m* is an exponent which can have a different value to *n* in Eq. (1). It should be noted that the traction becomes zero when the separation exceeds the critical value, i.e. $T_{n} = 0$ if $\delta _{n} \ge \delta ^{\mathrm {f}}_{n}$.

#### Kachanov damage type empirical model

In this model damage is assumed to influence the constitutive response and a single scalar damage parameter $\omega$ is introduced to incorporate the effect of damage. The damage parameter is assumed to evolve monotonically from 0 to 1, i.e. from an undamaged to a fully damaged state. Following Kachanov (1958) and Lemaitre and Chaboche (1994) we assume that the separation rate is a function of an effective traction $\bar{T}_{n}$, which is related to the normal traction $T_{n}$ and $\omega$ by

9 $$\begin{aligned} \bar{T}_{n} = \dfrac{T_{n}}{1-\omega }. \end{aligned}$$

The constitutive response is then simply obtained by replacing $T_{n}$ by $\bar{T}_{n}$ in Eq. (8) to give

10 $$\begin{aligned} \dot{\delta }_{n} = \dot{\delta }_{0} \left( \dfrac{T_{n}}{\left( 1-\omega \right) \, T_{0}}\right) ^{m}. \end{aligned}$$

**Fig. 3:**
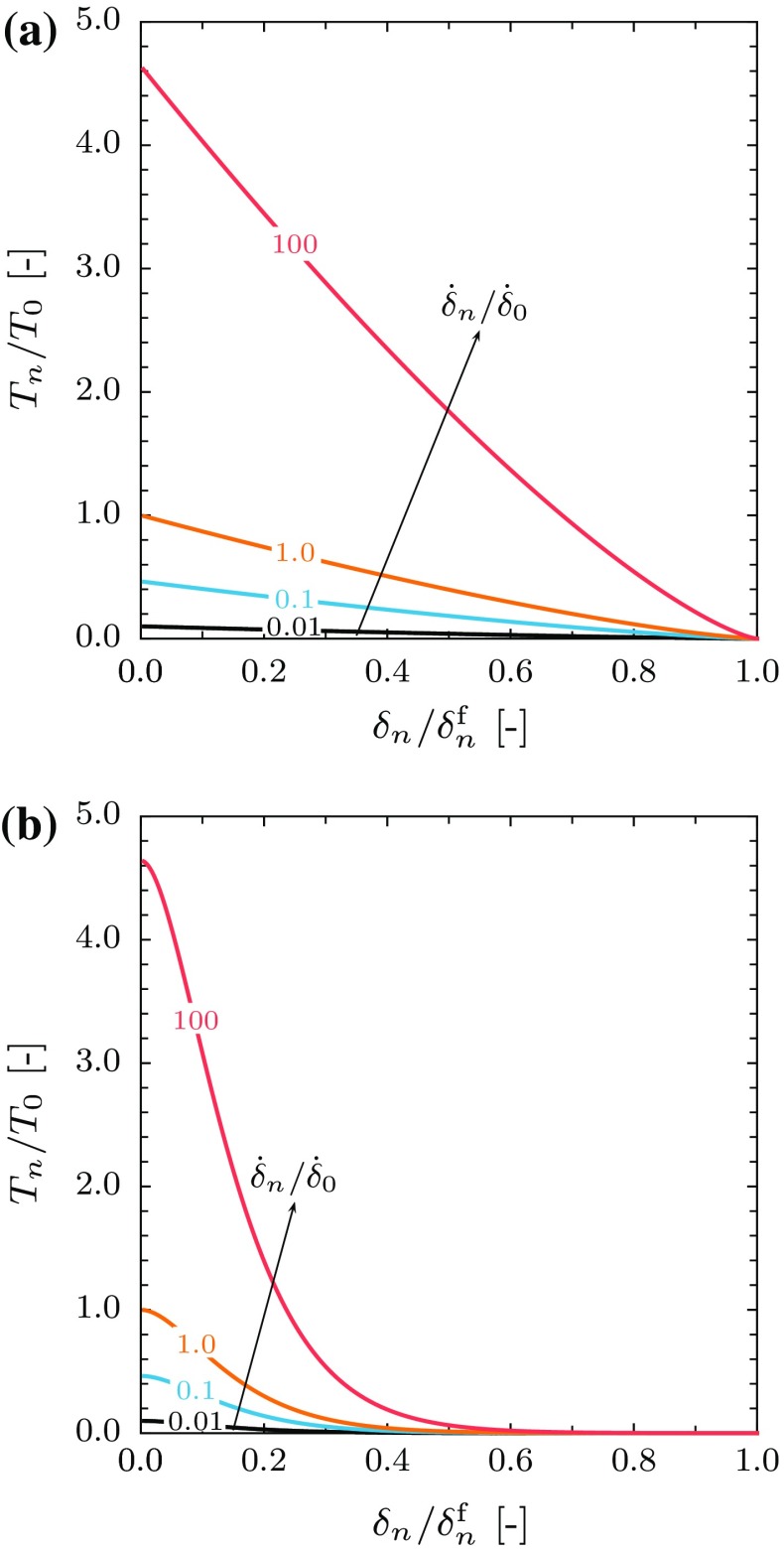
The Kachanov damage type model traction-separation rate law: **a** the linear damage model and **b** the exponential damage model. The rate sensitivity exponent is taken to be $m = 9$

The damage is assumed to be determined by the normal separation $\delta _{n}$, i.e. $\omega = \omega \left( \delta _{n} \right)$, and the damage evolution law may take different forms depending on the damage mechanism(s). In this study, we propose two different damage models, namely linear and exponential models. The different evolution laws are:

- The linear damage model:
  11 $$\begin{aligned} \omega = {\left\{ \begin{array}{ll} \dfrac{\delta _{n}-\delta _{n}^{\mathrm {c}}}{\delta _{n}^{\mathrm {f}}-\delta _{n}^{\mathrm {c}}} &{} \quad \text {if} \,\, \delta _{n} \ge \delta ^{\mathrm {c}}_{n}, \\ 0 &{} \quad \text {if} \,\, \delta _{n} < \delta ^{\mathrm {c}}_{n}. \end{array}\right. } \end{aligned}$$
- The exponential damage model:
  12 $$\begin{aligned} \omega = {\left\{ \begin{array}{ll} 1-\exp \left[ -\beta \, \left( \dfrac{\delta _{n}-\delta ^{\mathrm {c}}_{n}}{\delta ^{\mathrm {f}}_{n}-\delta ^{\mathrm {c}}_{n}} \right) \right] &{} \quad \text {if} \,\, \delta _{n} \ge \delta ^{\mathrm {c}}_{n}, \\ 0 &{} \quad \text {if} \,\, \delta _{n} < \delta ^{\mathrm {c}}_{n}. \end{array}\right. } \end{aligned}$$

where $\delta ^{\mathrm {c}}_{n}$ is the separation at which damage initiates (for separations less than this value $\omega = 0$ and the constitutive response is given by Eq. (1)), as before $\delta ^{\mathrm {f}}_{n}$ is the separation at failure and $\beta$ is a material parameter. It should be noted that for the exponential damage law the traction does not necessarily decrease smoothly to zero at failure but an abrupt response may result. Figure 3 below shows the traction-separation law for different separation rates for the case of linear and exponential damage laws.

#### Micromechanical based model

This model is based on the creep extension of Yalcinkaya and Cocks (2015) micromechanical damage zone model for ductile fracture described by Cocks et al. (2017), which are both derived from the creep cavitation model of Cocks and Ashby (1980). These models are based on the growth of an array of pores idealized as cylinders. The relation between the macroscopic traction and separation and the microscopic stress and strain is then obtained using classical bounding theorems.

**Fig. 4:**
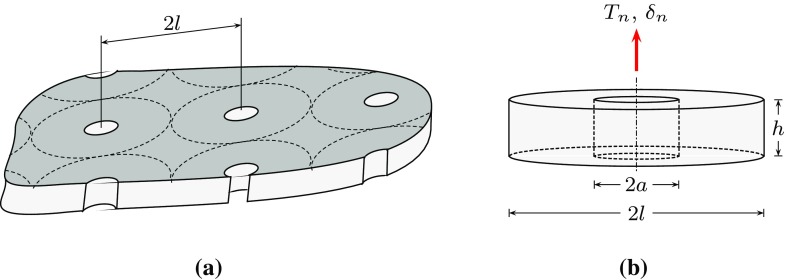
The micromechanical representation of the creep damage by pore growth: **a** pores of radius *r* and spacing 2*l* on a grain boundary subjected to microscopic stress state $\sigma _{ij}$ and the deformation is controlled by steady-state creep and **b** an idealization of a pore as a cylinder with height *h* and diameter equal to the pore to pore spacing 2*l* and the macroscopic normal traction $T_{n}$ and separation $\delta _{n}$

The radius and height of the pores at a given instant are denoted as *r* and *h*, respectively, and the mean spacing is 2*l*, see Fig. 4. Thus, the pores are characterized by their area fraction in the plane of the cavitated zone, i.e. by $f = (r/l)^2$. Further, the representative volume element is assumed to be fully constrained in the radial direction and the deformation is only controlled by the normal separation, i.e. $l = \mathrm {const.}$ and $\dot{h} = \dot{\delta }_{n}$. This simplification of the void profile captures the major features of the evolving geometry (such as area fraction of pores and pore aspect ratio) while allowing simple analytical expressions for the evolution of damage to be derived. More general forms of model are discussed by Cocks et al. (2017)—this is the simplest form of model of this class and it is directly equivalent in form to classical rate dependent cohesive zone models, including those described above. The resulting expression for the opening rate is

13 $$\begin{aligned} \dot{\delta }_{n} = \dot{\delta }_{0} \dfrac{1}{\bar{g}_{n}} \left( \dfrac{T_{n}}{\bar{g}_{n} \, T_{0}}\right) ^{m}, \end{aligned}$$

where $$\bar{g}_{n} = g_{n}(f)/g_{0}$$ and

14 $$\begin{aligned} g_{n} = \left[ \left( 1-f\right) ^2+\left( \frac{1}{\sqrt{3}} \, \ln \dfrac{1}{f}\right) ^2 \right] ^\frac{1}{2}. \end{aligned}$$

and $g_{0}$ is a parameter that can be used to provide a similar rupture time to the Kachanov models under the same stress level and the same value of the material parameters $\dot{\delta }_{0}$, $T_{0}$ and $\delta _{n}^{\mathrm {f}}$ (see “Appendix B”). The matrix material is incompressible, therefore the total rate of change in volume is equal to the rate of change in pore volume. Hence, the pore area fraction evolves at a rate

15 $$\begin{aligned} \dot{f} = \dfrac{\dot{\delta }_{n}}{h} \, \left( 1-f\right) . \end{aligned}$$

The initial pore area fraction and height are assumed to be $f_{0}$ and $h_{0}$, respectively. Further, the pores are assumed to coalesce and reach a complete failure when $f = f_{\mathrm {c}}$. The direct integration of Eq. (15) gives the separation at failure:

16 $$\begin{aligned} \int \limits ^{f_{\mathrm {c}}}_{f_{0}} \dfrac{1}{\left( 1-f\right) } \cdot \mathrm {d}f = \int \limits ^{h}_{h_{0}} \dfrac{1}{h} \cdot \mathrm {d}h \ \Longrightarrow \ \delta ^{\mathrm {f}}_{n} = h_{0} \left[ \dfrac{1-f_{0}}{1-f_{\mathrm {c}}}-1 \right] . \end{aligned}$$

**Fig. 5:**
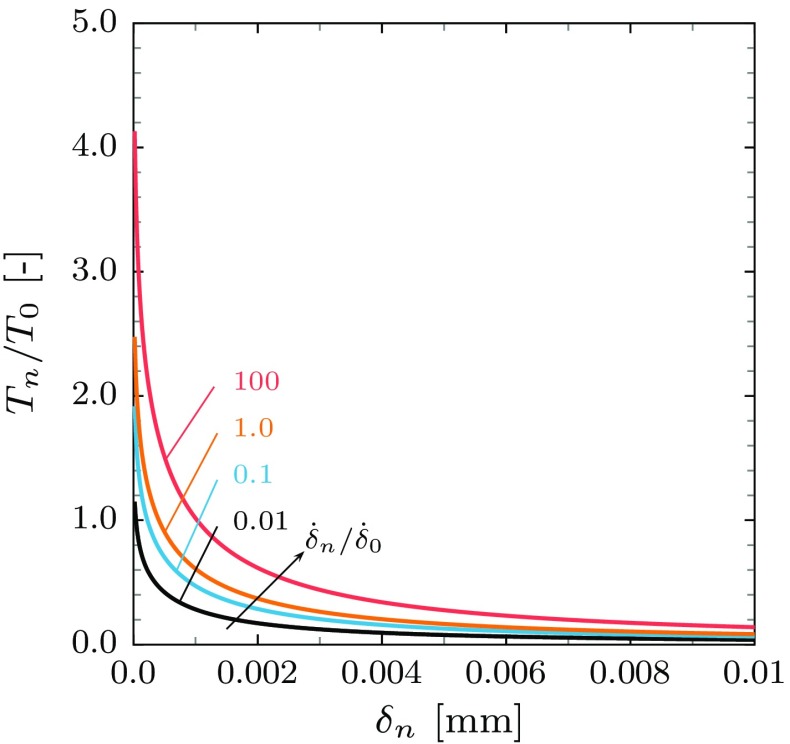
The micromechanical based model traction-separation rate law. The initial pore area fraction and height are assumed to be $f_{0}=0.01$ and $h_{0}=0.001$ $\mathrm {mm}$

The traction-separation rate relation for different separation rates is illustrated in Fig. 5.

## Creep crack growth in a double cantilever beam

**Fig. 6:**
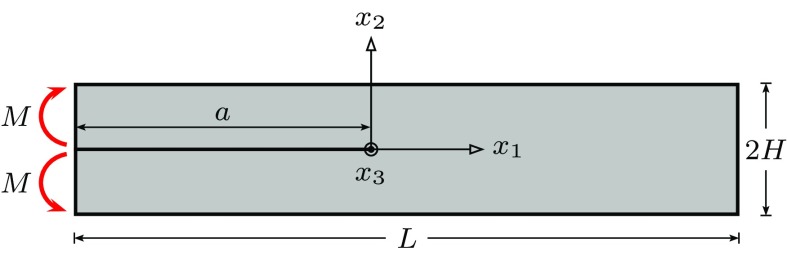
The schematic of the double cantilever beam specimen

In this section, pure mode-I creep crack growth in the DCB specimen shown in Fig. 6 is analysed. The length and height, of the specimen are denoted by *L* and 2*H*, respectively, and the crack length is denoted by *a*. Each arm of the specimen is subjected to a constant moment *M* per unit depth. We assume that the overall length $L \longrightarrow \infty$ and that the height of each arm $H \ll a$. Under these conditions $C^*$, remains constant as the crack grows, thus a steady state is eventually achieved in which the crack growth rate is constant. We focus on this steady state response. The objective is to obtain a mathematical description for the relationship between the far field loading and the local damage development within the damage zone and the crack growth rate under both plane stress and plane strain conditions.

**Fig. 7:**
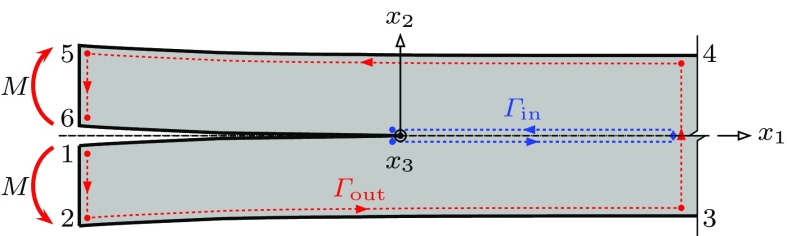
The definition of the *inner path* $\varGamma _{\mathrm {in}}$ and the *outer path* $\varGamma _{\mathrm {out}}$ that are used to evaluate the $C^*$-integral in the double cantilever beam specimen

In order to determine the relationship between the loading and fracture parameters, the path independence of the $C^*$-integral is used as discussed in Sect. 2. The $C^*$-integral is evaluated along the outer and inner paths indicated by the dashed lines and different colours in Fig. 7. Equating the values of $C^*$ determined from these two paths provides a relationship between the crack-tip opening rate and the applied load. There is a single characteristic geometric length scale for this problem, which we take as $\lambda = H/2$, and in the steady state the separation rate within the damage zone can be expressed as a function of $x_1/\lambda$, integrating this function as an element is convected towards the crack tip as the crack grows at constant velocity, allows the crack growth rate to be determined. We do not know the form of this function a priori, but to determine the crack growth rate we only need to determine a single quantity—the resulting integral, which can be determined from a single piece of information from a finite element analysis of the problem. The details of this process are given below.

### The $C^*$-integral in the outer path $\varGamma _{\mathrm {out}}$

$C^*$ in the outer path can be determined in a number of different ways—for example by direct evaluation of the integral of Eq. (5) or by determining the rate of change of the rate analogue of the total potential energy, $\Pi$, with crack length, where

17 $$\begin{aligned} \Pi =\int \limits _{V} \psi \cdot dV - \int \limits _{S}T_{i} \dot{u}_{i} \cdot \mathrm {d}S. \end{aligned}$$

We adopt the second of these approaches here. For an element of beam under bending the curvature rate is given by

18 $$\begin{aligned} \dot{\kappa } = \frac{2 \dot{\varepsilon }_{0}}{H} \left( \frac{2n+1}{2n} \ \frac{4M}{\sigma _{0} H^{2}} \right) ^{n} = \frac{2 \dot{\varepsilon }_{0}}{H} \eta \left( \frac{\eta M}{M_{0}} \right) ^{n}, \end{aligned}$$

where $M_{0} = \dfrac{2n}{2n+1} \, \dfrac{\sigma _{0} H^2}{4}$, and $\eta =1$ for plane stress and $\sqrt{3}/2$ for plane strain. For the DCB specimen of Fig. 7

19 $$\begin{aligned} C_{\mathrm {out}}^{*}&= \left. -\frac{\partial \Pi }{\partial a} \right| _{M} \nonumber \\&= 2 \left[ \frac{n}{n+1}\frac{2 \dot{\varepsilon }_{0}}{H} M_{0} \left( \frac{\dot{\kappa } H}{2 \dot{\varepsilon }_{0}} \right) ^{\frac{n+1}{n}} - M \frac{\partial \dot{\theta }}{\partial a} \right] \nonumber \\&= 2\left[ \frac{n}{n+1}\frac{2 \dot{\varepsilon }_{0}}{H} M_{0} \left( \frac{\dot{\kappa } H}{2 \dot{\varepsilon }_{0}} \right) ^{\frac{n+1}{n}} - M \dot{\kappa } \right] \nonumber \\&= \frac{2}{n+1} \frac{2 \dot{\varepsilon }_{0}}{H} M_{0} \left( \frac{\eta M}{M_{0}} \right) ^{n+1}. \end{aligned}$$

The factor of 2 arises because there are 2 beams and $\dot{\theta }$ is the rotation rate at the end of one of the beams. If we define a reference stress, such that

20 $$\begin{aligned} \sigma _{0} = \frac{2n+1}{2n} \frac{4\eta M}{ H^{2}}, \end{aligned}$$

and

21 $$\begin{aligned} C_{\mathrm {out}}^{*} = f_{n}\left( n \right) \dot{\varepsilon }_{0} \sigma _{0} \lambda , \end{aligned}$$

where $f_{n}\left( n \right) =\dfrac{4n}{\left( 2n+1\right) \left( n+1\right) }$ and $\lambda = H/2$ is the characteristic length scale for the DCB specimen.

### The $C^*$-integral in the inner path $\varGamma _{\mathrm {in}}$

Using the definition in Eq. (7), the $C^*$-integral in the inner path $\varGamma _{\mathrm {in}}$ can be written as

22 $$\begin{aligned} C^{*}_{\mathrm {in}} = \int \limits _{0}^{\dot{\delta }^{\mathrm {m}}_{n}} T_{n} \cdot \mathrm {d}\dot{\delta }_{n} = \int \limits _{0}^{\dot{\delta }^{\mathrm {m}}_{n}} d_{k} \, T_{0} \left( \dfrac{\dot{\delta }_{n}}{\dot{\delta }_{0}}\right) ^{\frac{1}{m}} \cdot \mathrm {d}\dot{\delta }_{n}, \end{aligned}$$

where the function $d_{k}$ depends on the form of interface model adopted, with *k* indicating the model, i.e. $\mathrm {s}\equiv$ simple, $\mathrm {kl}\equiv$ Kachanov linear, $\mathrm {ke}\equiv$ Kachanov exponential and $\mathrm {m}\equiv$ micromechanical models. For each of these models $d_{k}$ is given by

23 $$\begin{aligned} d_{\mathrm {s}}=1, \quad d_{\mathrm {ke}} \ \text {and} \ d_{\mathrm {ke}} = 1-\omega \quad \text {and} \quad d_{\mathrm {m}}= g^{\frac{m+1}{m}}_{n}. \end{aligned}$$

Apart for the simple model the integral requires a knowledge of the stress history experienced by each material point in the damage zone, which is not known a priori. For the simple model the integral of Eq. (22) can be readily determined:

24 $$\begin{aligned} C^{*}_{\mathrm {in}} = \int \limits _{0}^{\dot{\delta }^{\mathrm {m}}_{n}} T_{0} \left( \dfrac{\dot{\delta }_{n}}{\dot{\delta }_{0}}\right) ^{\frac{1}{m}} \cdot \mathrm {d}\dot{\delta }_{n} = \dfrac{2m}{m+1} \, \dot{\delta }_{0} \, T_{0} \left( \dfrac{\dot{\delta }^{\mathrm {m}}_{n}}{\dot{\delta }_{0}}\right) ^{\frac{m+1}{m}}. \end{aligned}$$

For all the remaining models $0 \le d_{k} \le 1$, and the resulting integral is therefore less than or equal to that given by Eq. (24). We assume that the integral for each model can be approximated by

25 $$\begin{aligned} C^{*}_{\mathrm {in}}= & {} \alpha _{k} \int \limits _{0}^{\dot{\delta }^{\mathrm {m}}_{n}} T_{0} \left( \dfrac{\dot{\delta }_{n}}{\dot{\delta }_{0}}\right) ^{\frac{1}{m}} \cdot \mathrm {d}\dot{\delta }_{n}\nonumber \\= & {} \alpha _{k} \, \dfrac{2m}{m+1} \, \dot{\delta }_{0} \, T_{0} \left( \dfrac{\dot{\delta }^{\mathrm {m}}_{n}}{\dot{\delta }_{0}}\right) ^{\frac{m+1}{m}}, \end{aligned}$$

where the subscript *k* again identifies the model and $\alpha _{k}$ falls in the range $0 \le \alpha _{k} \le 1$.

### The crack tip opening displacement rate

Equating the values of $C^*$ given by the inner and outer contours, i.e. Eqs. (21) and (25), allows the crack tip opening displacement rate $\dot{\delta }^{\mathrm {m}}_{n}$ to be expressed as a function of the applied loading. Equating $T_{0}$ to $\sigma _{0}$ gives

26 $$\begin{aligned} \dot{\delta }_{n}^{\mathrm {m}} = \dot{\delta }_{0} \left( \frac{q\left( n,m\right) \, \phi _{0}}{\alpha _{k}} \right) ^{\frac{m}{m+1}}, \end{aligned}$$

where $\phi _0 = \dfrac{\dot{\varepsilon }_{0} \, \lambda }{\dot{\delta }_{0}} = \dfrac{\dot{\varepsilon }_{0} \, H}{2 \dot{\delta }_{0}}$ is the ratio of geometric to material length scales for the problem and $g\left( n,m\right) = f_{n}\left( n\right) \cdot \dfrac{m+1}{2 m} = \dfrac{2 n \left( m+1\right) }{m\left( 2n+1\right) \left( n+1\right) }$, which for $n = m$ reduces to $q\left( n,n\right) = q_{n}\left( n\right) = \dfrac{2}{2n+1}$.

### The analysis of a steadily propagating crack

**Fig. 8:**
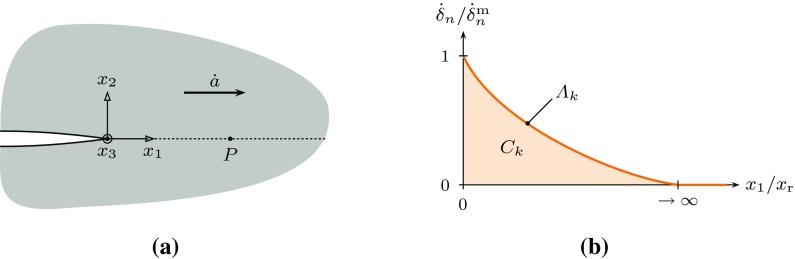
The schematics of a steadily propagating crack in viscous solid: **a** a material point *P* at distance $x_{1}$ from the moving crack tip and **b** the definition of the $\varLambda _{k}$ and $C_{k}$ dimensionless functions for point *P*

As noted earlier, under a constant applied moment the crack velocity will, after an initial transient, achieve a steady state, in which it reaches a constant value $\dot{a}$. We consider a coordinate system that moves with the crack tip. A material element such as *P* in Fig. 8a then moves along the $x_1$-direction at a rate (Cocks and Ashby 1981)

27 $$\begin{aligned} \dfrac{\mathrm {d} x_1}{\mathrm {d} t} = -\dot{a}. \end{aligned}$$

The are two characteristic length scales in this problem, the geometric length scale $\lambda$ and the material length scale $\dot{\delta }_{0} / \dot{\varepsilon }_{0}$. We can therefore write the separation rate in the form

28 $$\begin{aligned} \dot{\delta }_{n} = \dot{\delta }^{\mathrm {m}}_{n} \cdot \varLambda _{k} \left( \dfrac{x_1}{\lambda } \right) = \dot{\delta }^{\mathrm {m}}_{n} \cdot \varLambda _{k} \left( \bar{x}_1\right) , \end{aligned}$$

where $\varLambda _{k}$ is a dimensionless function that depends on the interface model whose detailed form depends on $\phi _0$, the ratio of geometric and material length scales, as shown in Fig. 8b. For each of the models described in Sect. 2, failure of an element occurs when the separation across the damage zone reaches a critical value, $\delta ^{\mathrm {f}}_{n}$. Integrating the displacement rate as an element is convected towards the crack tip gives

29 $$\begin{aligned} \delta ^{\mathrm {f}}_{n}&= \int \limits ^{t_{\mathrm {f}}}_{0} \dot{\delta }_{n} \cdot \mathrm {d}t = -\int \limits ^{0}_{\infty } \dot{\delta }_{n} \cdot \dfrac{\mathrm {d}x_1}{\dot{a}} \nonumber \\&= \dfrac{\dot{\delta }^{\mathrm {m}}_{n} \, \lambda }{\dot{a}} \, \int \limits ^{\infty }_{0} \varLambda _{k}\left( \bar{x}_1\right) \cdot \mathrm {d} \bar{x}_1 \nonumber \\&= \dfrac{\dot{\delta }^{\mathrm {m}}_{n} \,\lambda }{\dot{a}} \, C_{k} \left( \phi _0,n,m \right) , \end{aligned}$$

where we have used Eq. (27) to substitute for $\mathrm {d}t$ and Eq. (28) to substitute for $\dot{\delta }_{n}$. The dimensionless function $C_{k} \left( \phi _0,n,m \right)$ is only a function of $\phi _0$, *n*, *m* and the detailed form of model for the damage zone. Substituting for $\dot{\delta }^{\mathrm {m}}_{n}$ using Eq. (24) gives the steady state crack velocity.

30 $$\begin{aligned} \overset{\triangledown }{a}&= \frac{\dot{a}}{\dot{\varepsilon }_{0} \lambda } = q\left( n,m \right) ^{\frac{m}{m+1}} \frac{\phi _{0}^{-\frac{1}{m+1}}}{\bar{\delta }_{n}^{\mathrm {f}}} \frac{C_{k}\left( \phi _{0},n,m \right) }{\alpha _{k}^{\frac{m}{m+1}}} \nonumber \\&= q\left( n,m\right) ^{\frac{m}{m+1}} \frac{\phi _{0}^{-\frac{1}{m+1}}}{\bar{\delta }_{n}^{\mathrm {f}}} \hat{C}_{k}\left( \phi _{0},n,m \right) . \end{aligned}$$

where $\bar{\delta }^{\mathrm {f}}_{n} = \delta ^{\mathrm {f}}_{n}/\lambda$. We can express this relationship in a number of different forms. An alternative form that can be used to provide some insight into the material response is:

31 $$\begin{aligned} \dot{a} = \frac{A^{\frac{1}{m+1}} \lambda }{\delta _{n}^{\mathrm {f}}} \left[ \frac{m+1}{2m} C^{*} \right] ^{\frac{m}{m+1}} \hat{C}_{k}\left( \phi _{0},n,m\right) \end{aligned}$$

where $A = \dot{\delta }_{0}/\sigma _{0}^{m}$ is a material constant for the damage zone [see Eq. (8)]. The form of this equation might suggest that the crack growth rate is a function of $C^*$, for a given value of $\delta ^{\mathrm {f}}_{n}$. This is only true if $\hat{C}_{k}$ is only a function of *n* and *m* or $\phi _0$ is constant for the range of conditions of interest. Note that for the DCB specimen of Fig. 7 and $m = n$

32 $$\begin{aligned} \phi _{0}=\frac{B}{A} \lambda = \frac{B}{A}\frac{H}{2} \end{aligned}$$

where $B = \dot{\varepsilon }_{0}/\sigma _{0}^{m}$ is a material property. Then for a series of experiments in which the geometry is kept constant we would expect $\dot{a}$ to be proportional to ${C^{*}}^{\frac{m}{m+1}}$, but the constant of proportionality could be different for a different choice of beam height *H*. For more general cracked geometries $\sigma _{0}$, $\dot{\varepsilon }_{0}$ and $\lambda$ change as a crack grows and therefore $\phi _{0}$ also changes. This needs to be taken into account in any model and description of the crack growth process. We consider this feature of the response further below.

In order to determine the crack growth rate we need to evaluate the quantity $\hat{C}_{k}$. We can determine this using the finite element method. We need not determine the distributions $d_{k}$ and $\varLambda _{k}$ ahead of the crack tip. We can determine directly by equating the numerically determined crack velocity with the prediction of Eq. (30).

### Numerical implementation of the governing equations

The initial-boundary value problem described in Sect. 3 is numerically solved using the FE (Finite Element) code ABAQUS (Abaqus 2016). A nonlinear quasi-static analysis is used for the initial loading, and a nonlinear visco analysis is used for the creep crack propagation analysis. In the visco analysis implicit time integration is used to solve the FE equations and mixed implicit/explicit integration is used for the integration of the creep and damage zone equations. The FE analysis requires the solution for an elastic/creep constitutive law in the bulk an elastic-rate dependent opening model for the damage zone. Elastic constitutive components have been added to the constitutive relationships of Eqs. (1), (8), (10) and (13), with the values of the elastic components chosen to have limited influence on the computed results.

**Fig. 9:**
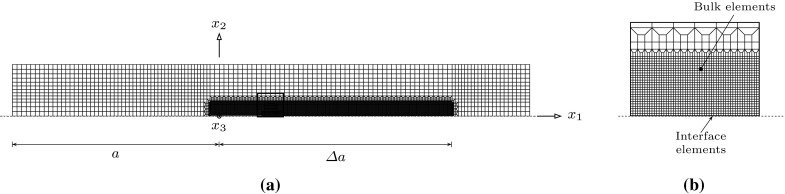
The finite element mesh of the double cantilever beam specimen: **a** the mesh of the whole geometry; and **b** mesh details along the middle of the specimen where the cohesive elements are inserted along the crack propagation path

The geometry of the double cantilever beam (DCB) specimen shown in Fig. 6 is discretised, and a typical finite element mesh is shown in Fig. 9. Only one half of the specimen is analysed due to the symmetry of the problem. The dimensions are taken as $L = 100$ $\mathrm {mm}$, $H = 10$ $\mathrm {mm}$, and $B = 1$ $\mathrm {mm}$. The initial crack is assumed to be $a = 40$ $\mathrm {mm}$, and the crack propagation is studied over a length of $\varDelta a = 50$ $\mathrm {mm}$. The 4-node reduced integration bilinear plane stress and strain elements (CPS4R and CPE4R) are used in the discretisation for plane stress and strain conditions, respectively. A 4-node two-dimensional linear damage zone element was implemented in ABAQUS using the user-defined subroutine UEL. The details of the Finite Element implementation are provided in “Appendix A”. The Finite Element model is divided into two regions, in which the bulk and damage zone elements are defined. The damage zone elements are inserted along the crack propagation path, i.e. along $a \le x_1 \le L$ and $x_2 = 0$, and the bulk elements are defined elsewhere. The top faces of the damage zone elements are attached to the bulk elements, see Fig. 9b.

The damage zone elements are modelled with zero initial thickness such that the top and bottom face nodes coincide. The mesh has 11,634 elements, of which 11,279 are bulk elements and 355 are damage zone elements. A uniform refined element region is created adjacent to the crack and its propagation for controlling the interface element length $l_{\mathrm {inte}}$. A convergence study on the mesh refinement was carried out for different values of $\phi _{0}$ and interface parameters $\bar{\delta }_{n}^{\mathrm {f}}=0.004$, $\beta =1.0$, $f_{0}=0.01$ and $f_{\mathrm {c}}=0.91$. We found that an interface element length of $l_{\mathrm {inte}} = 0.1$ $\mathrm {mm}$ is necessary to obtain converged solutions for the range $\phi _{0} \in \left[ 10^{-5}-10^{4}\right]$. The interface stiffness $K_{n} = K_{t} = 10^{6}$ $\mathrm {MPa}\cdot \mathrm {mm}$ is selected such that the elastic deformation is negligible (see “Appendix A”).

**Fig. 10:**
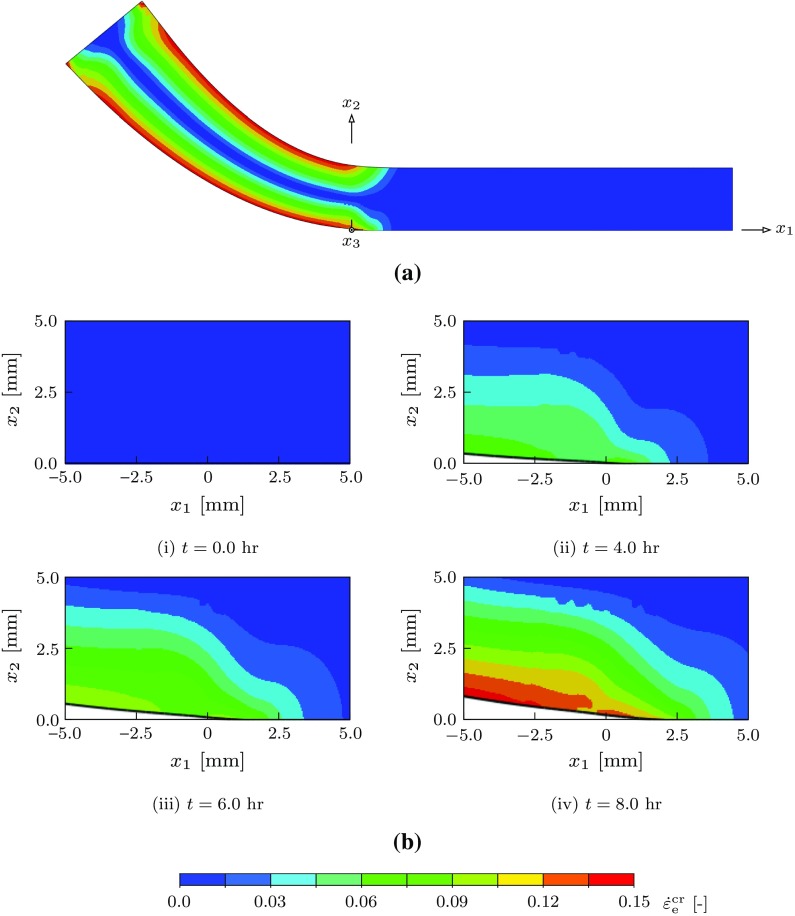
The Distribution of the effective creep strain $\dot{\varepsilon }_{\mathrm {e}}^{\mathrm {cr}}$ for the parameters $n=m=9$, $\phi _{0}=1.0$ and $\bar{\delta }^{\mathrm {f}}_{n}=0.004$: **a** the deformed DCB specimen at time $t = 8$ $\mathrm {h}$; and **b** the propagating crack tip at different instants of time

The numerical analysis was performed for different combinations of the dimensionless parameters defined above to confirm that the functional form of Eq. (30) is valid. (The model parameters are chosen in such a way that the dimensionless parameters are controlled.) The relative normal separation displacement, $\varDelta u_2$, between each pair of initially coincident nodes in the interface ($x_2 = 0$) is computed and recorded during the analysis. The crack tip position, $x_{\mathrm {tip}}$, is defined by $\varDelta u_2 = \delta _{n}^{\mathrm {f}}$, and the crack tip velocity is determined using forward differencing as

33 $$\begin{aligned} v^{p}_\mathrm {tip} = \left. \dfrac{\mathrm {d} x_\mathrm {tip}}{\mathrm {d} t} \right| _{t_{p}} = \dfrac{x^{p+1}_\mathrm {tip}-x^{p}_\mathrm {tip}}{\varDelta t_{p}} \end{aligned}$$

where indices *p* and $p+1$ denote variable values at instants $t_{p}$ and $t_{p+1}$, respectively, and $\varDelta t_{p}=t_{p+1}-t_{p}$ is the time increment. Further, the steady crack velocity, $\dot{a}$, is computed by taking the average velocity over the steady propagation period.

## Results and discussion

### The crack growth

Several analyses have been performed for different combinations of the dimensionless parameters and damage zone properties. The crack tip position, transient and steady state crack propagation velocity have been obtained for all the combinations. For the simple interface model the parameters $n=m=9$, $\phi _{0}=1.0$ and $\bar{\delta }^{\mathrm {f}}_{n}=0.004$ are used to illustrate the different results. We first describe the results under plane stress conditions.

Figure 10i–iv show the distribution of the effective creep strain $\dot{\varepsilon }_{\mathrm {e}}^{\mathrm {cr}}$ at four different instants and crack velocities. At $t = 0$ the creep deformation is zero everywhere and elastic deformation prevails. As time passes ($t > 0$) creep deformation evolves in the bulk, initially primarily in the vicinity of the crack tip, as well as damage along the interface, leading eventually to crack growth when the critical opening is achieved at the crack tip.

**Fig. 11:**
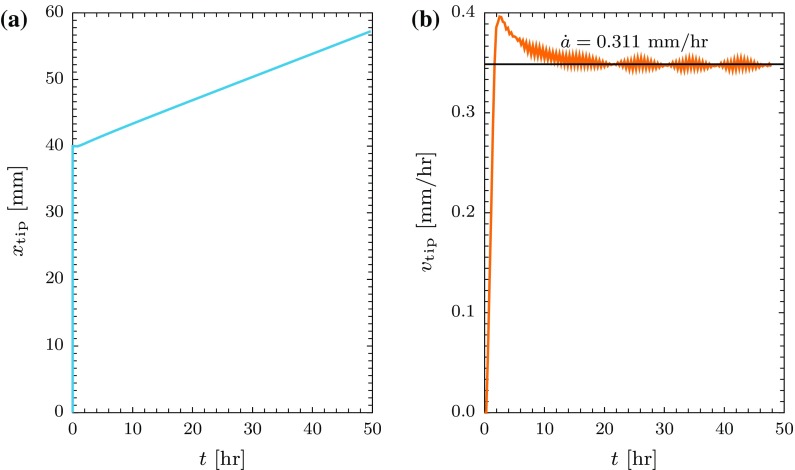
Crack propagation results for $n=m=9$, $\phi _{0}=1.0$ and $\bar{\delta }^{\mathrm {f}}_{n}=0.004$: **a** crack tip position $x_{\mathrm {tip}}$ versus time *t*; and **b** crack tip velocity $v_{\mathrm {tip}}$ versus time *t*

Figure 11a, b show the crack tip position and velocity as functions of time, respectively. The plots show that the crack starts to propagate slowly and accelerates to a high velocity and after a short time (10 $\mathrm {h}$) a lower steady state velocity is achieved. In this case a steady velocity of $\dot{a} = 0.311$ $\mathrm {mm}/\mathrm {h}$ is obtained. The result shows that the transient velocity is higher than the steady state velocity suggesting that the stress at the crack tip is initially high due to the elastic deformation and as the crack advances the creep deformation dominates where the stress relaxes leading to a slower propagation rate. Additionally the damage ahead of the crack tip is fully developed during both transient and steady propagation. The other scenario is when the damage is not fully developed during the transient stage which may lead to a slower propagation before reaching a steady state where a fully developed damage zone is achieved.

### The $C_{s}$-function

It proves instructive to concentrate initially on the response for the simple damage zone model of Sect. 2.3.1. The Finite Element analysis is used to determine the steady crack velocity $\dot{a}$ and then for a given set of input parameters the $C_{s}$-function can be evaluated from Eq. (30):

34 $$\begin{aligned} \hat{C}_{k} = \frac{\overset{\triangledown }{a} \, \bar{\delta }_{n}^{\mathrm {f}} \, \phi _{0}^{\frac{1}{m+1}}}{g\left( n,m\right) ^{\frac{m}{m+1}}}. \end{aligned}$$

The appropriateness of the dimensionless analysis has been examined using the same set of dimensionless parameters with different model parameters, e.g. the same value of $\phi _{0}$ with different combinations of$\dot{\varepsilon }_{0}$, *H* and $\dot{\delta }_{0}$.

### The physical limits and the validity of the framework

Before evaluating the computational results in detail it is instructive to examine the response in the limits of small and large $\phi _{0}$. The first extreme is when the interface is very stiff in comparison with the bulk material (the bulk material creeps faster than the interface, i.e. $\dot{\varepsilon }_{0} \gg \dot{\delta }_{0}/\lambda$ and $\phi _{0}\rightarrow \infty$). The other extreme occurs when the interface creeps faster than the bulk material, i.e. the interface is very compliant ($\dot{\varepsilon }_{0} \ll \dot{\delta }_{0}/\lambda$ and $\phi _{0}\rightarrow 0$). In this analysis we consider the simple damage zone model of Sect. 2.3.1.

When an interface is very stiff in comparison with the bulk material the deformation along the interface is negligible and it does not influence the stress state in the body. The tractions seen by the damage zone are determined by the stress distribution in the bulk material, and can be expressed in terms of the $C^{*}$-integral (provided the damage zone is small compared to the region in which the HRR field dominates; Hutchinson 1968; Rice and Rosengren 1968). The HRR stress field is defined as

35 $$\begin{aligned} \sigma _{ij} = \sigma _{0} \, \left[ \dfrac{C^{*}}{\dot{\varepsilon }_{0} \, \sigma _{0} \, I_{n} \, r} \right] ^{\frac{1}{n+1}} \, \tilde{\sigma }_{ij}\left( n,\theta \right) , \end{aligned}$$

where $I_{n}$ is an integration constant that depends on *n* and $\tilde{\sigma }_{ij}$ is a dimensionless function of *n* and $\theta$. The values of these parameters are given for the cases of plane stress and plane strain conditions by Hutchinson (1968). It follows that the normal traction along the interface is given by

36 $$\begin{aligned} T_{n} = \sigma _{0} \, \left[ \dfrac{C^{*}}{\dot{\varepsilon }_{0} \, \sigma _{0} \, I_{n} \, r }\right] ^{\frac{1}{n+1}} \, \tilde{\sigma }_{\theta }\left( n,0\right) , \end{aligned}$$

In this analysis, we limit ourself to the case of $T_{0} = \sigma _{0}$ and $m = n$. Therefore the opening separation rate for the simple model is evaluated from Eq. (8) as

37 $$\begin{aligned} \dot{\delta }_{n} = \dot{\delta }_{0} \, \left[ \dfrac{C^{*}}{\dot{\varepsilon }_{0} \, \sigma _{0} \, I_{n} \, r }\right] ^{\frac{n}{n+1}} \, \tilde{\sigma }_{\theta }\left( n,0\right) ^{n}. \end{aligned}$$

The critical opening separation is determined by integrating the separation rate, in a similar way to in Eq. (8), as

38 $$\begin{aligned} \delta _{n}^{\mathrm {f}}&= \int \limits _{0}^{\infty } \dot{\delta }_{n} \cdot \dfrac{\mathrm {d}x_1}{\dot{a}} = \int \limits _{0}^{r_{\mathrm {c}}} \dot{\delta }_{n} \cdot \dfrac{\mathrm {d}r}{\dot{a}} \nonumber \\&= \left( n+1 \right) \, \dfrac{\dot{\delta }_{0}}{\dot{a}} \, \left[ \dfrac{C^{*}}{\dot{\varepsilon }_{0} \, \sigma _{0} \, I_{n} }\right] ^{\frac{n}{n+1}} \, r_{\mathrm {c}}^{\frac{1}{n+1}} \, \tilde{\sigma }_{\theta }\left( n,0\right) ^{n}. \end{aligned}$$

where $r = x_{1}$ at $\theta = 0$ and $r_{\mathrm {c}}$ is the size of the fracture zone which is very small in the case of stiff interface ($r_{\mathrm {c}} \rightarrow 0$). Rearrangement of Eq. (38) gives the dimensionless velocity as

39 $$\begin{aligned} \overset{\triangledown }{a} = \left( n+1 \right) \dfrac{\bar{r}_{\mathrm {c}}^{\frac{1}{n+1}}}{\phi _{0} \, \bar{\delta }_{n}^{\mathrm {f}}} \, \left[ \dfrac{f_{n}\left( n \right) }{I_{n}}\right] ^{\frac{n}{n+1}} \, \tilde{\sigma }_{\theta }\left( n,0\right) ^{n}, \end{aligned}$$

where $\bar{r}_{\mathrm {c}} = r_{\mathrm {c}} / \lambda$. By comparing this equation with Eq. (30), the $C_{\mathrm {s}}$-function for the case stiff interface becomes

40 $$\begin{aligned} C_{\mathrm {s}} = \left( n+1 \right) \, \bar{r}_{\mathrm {c}}^{\frac{1}{n+1}} \, \left[ \dfrac{2n}{n+1} \cdot \dfrac{1}{\phi _{0} \, I_{n}}\right] ^{\frac{n}{n+1}} \, \tilde{\sigma }_{\theta }\left( n,0\right) ^{n}. \end{aligned}$$

For the case of $n=m=9$ and $\theta = 0$, the integration constants are $I_{9} \approx 3.025$ and $\tilde{\sigma }_{\theta }\left( 9,0\right) \approx \tilde{\sigma }_{\theta }\left( 13,0\right) \approx 1.2$ for the case of plane stress and $I_{9} \approx 4.6$ and $\tilde{\sigma }_{\theta }\left( 9,0\right) \approx \tilde{\sigma }_{\theta }\left( 13,0\right) \approx 2.6$ for the case of plane strain (Hutchinson 1968). Thus, the $C_{\mathrm {s}}$-functions for the cases of plane stress and plane strain conditions are $C_{\mathrm {s}} = 45.6 \cdot \bar{r}_{\mathrm {c}}^{0.1} \cdot \phi _{0}^{-0.9}$ and $C_{\mathrm {s}} = 7.1 \cdot 10^{4} \cdot \bar{r}_{\mathrm {c}}^{0.1} \cdot \phi _{0}^{-0.9}$, respectively.

The other limit is when the interface is too compliant in comparison with the bulk material which can be regarded as rigid. Hence, in the case of an infinite DCB specimen, the equilibrium between the applied moment and the traction along the infinite damage zone suggests that the traction will tend to zero and there will be no crack propagation. on the other hand, when the specimen is finite a non zero traction along the finite damage zone. The deformation along the damage zone can directly be related to the angular deflection at the end of the beam. Thus, the separation at the crack tip is obtained as

41 $$\begin{aligned} \delta _{n}^{\mathrm {f}} = 2 \, \left( L-a\right) \theta = 2 \, W \, \theta , \end{aligned}$$

where $W=L-a$ is the length of remaining ligament during steady state propagation. The opening displacement at the tip of the propagating crack is constant and equal to the critical value $\delta _{n}^{\mathrm {f}} = 0$, therefore $\dot{\delta }_{n} = 0$, and

42 $$\begin{aligned} \dot{a} = W \, \dfrac{\dot{\theta }}{\theta }. \end{aligned}$$

Similarly, the separation at the crack tip can be written in this form

43 $$\begin{aligned} \dot{\delta }_{n}^{\mathrm {m}} = 2 \, W \dot{\theta }. \end{aligned}$$

The separation rate in the damage zone is given by

44 $$\begin{aligned} \dot{\delta }_{n} = \left[ 1-\dfrac{x_1}{W}\right] \cdot \dot{\delta }_{n}^{\mathrm {m}}. \end{aligned}$$

Now the balance by the internal and external work rates gives

45 $$\begin{aligned} 2 \, M \, \theta = \int \limits _{0}^{W} T_{n} \, \dot{\delta }_{n} \cdot \mathrm {d}x_{1} = \int \limits _{0}^{W} \dot{\delta }_{0} \, T_{0} \left( \dfrac{\dot{\delta }_{n}}{\dot{\delta }_{0}}\right) ^{\frac{n+1}{n}} \cdot \mathrm {d}x_{1}. \end{aligned}$$

Introducing Eq. (43) and the opening separation rate for the simple model in Eq. (8) we obtain the separation at the crack tip as

46 $$\begin{aligned} \dot{\delta }_{n}^{\mathrm {m}} = \dot{\delta }_{0} \, \left[ \dfrac{2n+1}{n} \, \dfrac{M}{\sigma _{0} \, W^{2}} \right] ^{n}. \end{aligned}$$

The crack velocity is determined from Eqs. (41), (42), (43) and (46) and using the definition of $\sigma _{0}$ in Eq. (20) as

47 $$\begin{aligned} \dot{a}= \dfrac{\dot{\delta }_{0}}{W^{2n-1} \, \delta _{n}^{\mathrm {f}}} \, \left[ \dfrac{2}{\eta } \right] ^{n}. \end{aligned}$$

Scaling of Eq. (47) gives the dimensionless velocity as

48 $$\begin{aligned} \overset{\triangledown }{a} = \dfrac{1}{\phi _{0} \, \bar{W}^{2n-1} \, \bar{\delta }_{n}^{\mathrm {f}}} \, \left[ \dfrac{2}{\eta } \right] ^{n} \end{aligned}$$

By comparing this equation with Eq. (30), the $C_{\mathrm {s}}$ function for the case compliant interface becomes

49 $$\begin{aligned} C_{\mathrm {s}} = \dfrac{1}{q_{n}^{\frac{n}{n+1}} \, \bar{W}^{2n-1}} \, \left[ \dfrac{2}{\eta }\right] ^{n} \, \phi _{0}^{-\frac{n}{n+1}}. \end{aligned}$$

$\bar{W}$ is computed from the finite element analysis as the remaining ligament length when a crack reaches a steady state propagation. Hence, for the case of $n=m=9$ and using the computationally obtained average value $\bar{W}\approx 0.8$, the $C_{\mathrm {s}}$-functions for plane stress and plane strain conditions are $C_{\mathrm {s}} = 10.5 \times 10^{-15} \cdot \phi _{0}^{-0.9}$ and $C_{\mathrm {s}} = 38.3 \times 10^{-15} \cdot \phi _{0}^{-0.9}$, respectively.

Another limitation comes from the time scale of the crack propagation as mentioned in Sect. 2.1. $C^{*}$ represents the near crack tip field when a crack propagates slowly. As the crack velocity increases elastic deformation becomes increasingly important in the vicinity of the crack tip and a zone in which both elastic and creep deformation determines the response becomes increasingly significant. If this zone becomes comparable in size to the damage zone, then $C^{*}$ can no longer be used as a parameter for characterization of the near tip filed and damage growth process. Cocks and Julian (1991) studied this limit and proposed conditions for the dominance of $C^{*}$. They demonstrate that $C^{*}$ controls crack growth provided the following condition is satisfied

50 $$\begin{aligned} \overset{\triangledown }{a} = f_{n}\left( n\right) ^{\frac{1}{n+1}} \, \dfrac{Z\left( n\right) }{\sigma _{0} / E } \, \bar{r}_{\mathrm {c}}^{\frac{2}{n+1}} \end{aligned}$$

where *E* is Young’s modulus and $Z\left( n\right) =\left( n-1\right) \, {I_{n}}^{\frac{n-1}{n+1}}$. Using this condition we derive a condition for $C_{\mathrm {s}}$ function by comparing Eq. (50) with Eq. (30) as

51 $$\begin{aligned} C_{\mathrm {s}} \le \dfrac{2n}{n+1} \, f_{n}\left( n\right) ^{\frac{1}{n+1}} \, Z\left( n\right) \, \dfrac{\bar{r}_{\mathrm {c}}^{\frac{2}{n+1}} \, \bar{\delta }_{n}^{\mathrm {f}}}{\sigma _{0} / E } \, \, \phi _{0}^{\frac{1}{n+1}}. \end{aligned}$$

This expression implies that for particular values of $\bar{\delta }_{n}^{\mathrm {f}}$ and $\sigma _{0}/E$ there is a maximum velocity for which $C^{*}$ is a valid measure. Thus, for the case of $n=m=9$, the valid $C_{\mathrm {s}}$-function for plane stress and plane strain conditions are $C_{\mathrm {s}} \le 4.28 \cdot \dfrac{\bar{r}_{\mathrm {c}}^{0.2} \, \bar{\delta }_{n}^{\mathrm {f}}}{\sigma _{0}/E} \cdot \phi _{0}^{0.1}$ and $C_{\mathrm {s}} \le 6.0 \cdot \dfrac{\bar{r}_{\mathrm {c}}^{0.2} \, \bar{\delta }_{n}^{\mathrm {f}}}{\sigma _{0}/E} \cdot \phi _{0}^{0.1}$, respectively.

Elasticity is only relevant in the computational models and this relationship can be used to assess whether the conditions employed in the FE models are consistent with the assumptions of the analytical model presented in Sect. 2. We need to be careful, however, when using this expression. It is derived from analyses in which damage development is assumed to not influence the near tip fields. As illustrated above, the size of the damage zone increases with decreasing $\phi _{0}$ and for small $\phi _{0}$ the near tip fields given by the classical continuum analysis are no longer valid. The relationship of Eq. (49) is therefore only valid in the limit of large $\phi _{0}$ where the development of damage has limited effect on the crack tip fields. It is also important to emphasise here that although, the HRR field is no longer valid for small $\phi _{0}$, $C^{*}$ is still a valid parameter for characterizing crack growth.

**Fig. 12:**
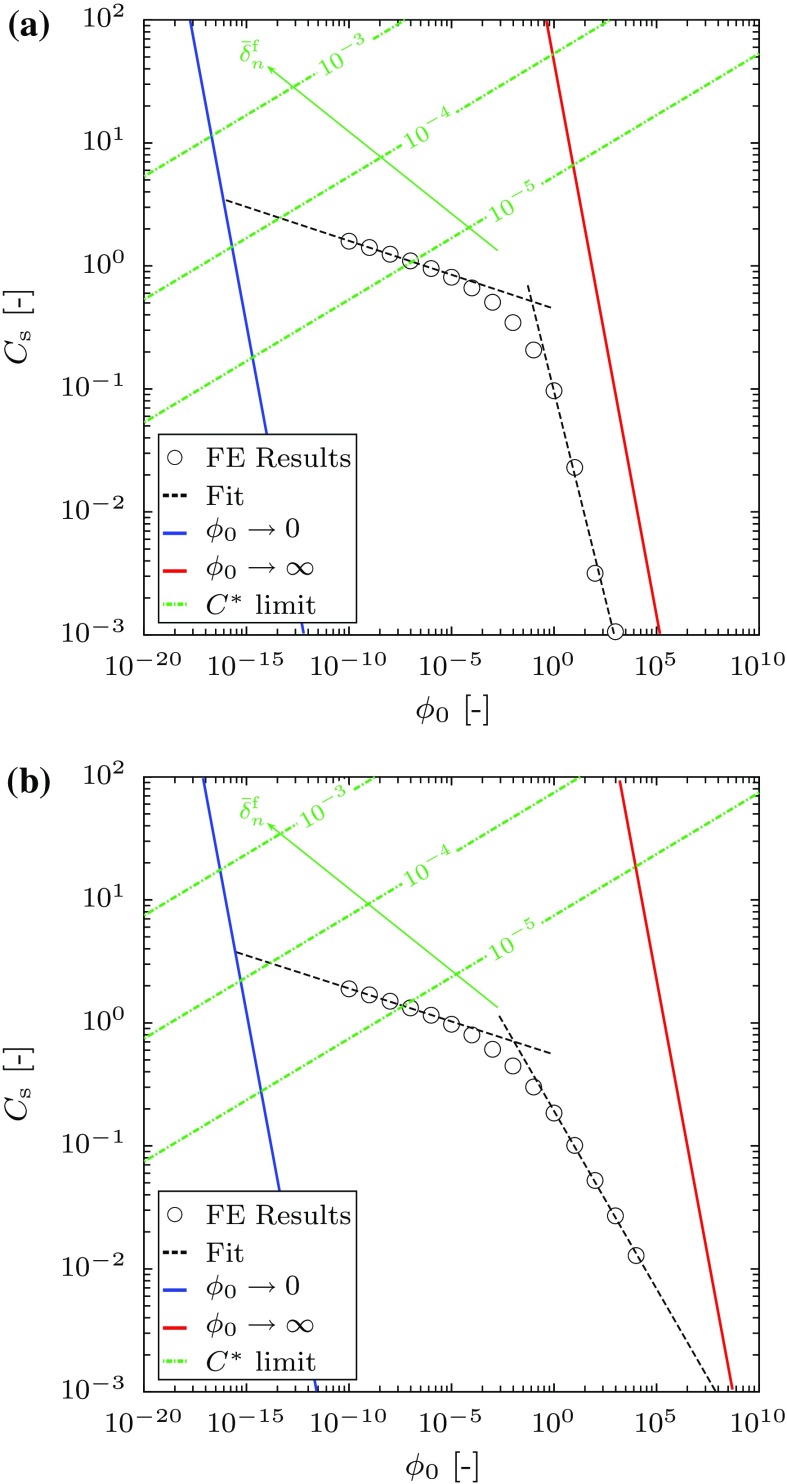
The relation between $C_{\mathrm {s}}$-function and $\phi _{0}$ parameter and the physical limits in the case of $n = m = 9$: **a** plane stress conditions; **b** plane strain conditions. The *red* and *blue lines* represent the compliant and stiff limits, respectively, the *green dash-dot lines* represent the $C^{*}$ validity limit for different dimensionless separation at failure $\bar{\delta }_{n}^{\mathrm {f}}$, and the *dashed lines* show the power law fit

In order to evaluate the proposed framework, $C_{s}$ has been determined from (34) for $\phi _{0}$ in the range [$10^{-10}$, $\times 10^{5}$] and compared with the limiting results presented above. The rate sensitivity parameters are taken to be $n = m = 9$. Figure 12a, b show the relationship between $C_{s}$ and $\phi _{0}$ for plane stress and strain conditions, respectively.

Over the range of the data, the results can be fit using two separate power-law relations. Under plane stress conditions this relation is $C_{s} = 0.45 \, \phi _{0}^{-0.06}$ over the range of values $\phi _{0} \in [10^{-10}, 8\times 10^{-2}]$ and $C_{s} = 0.09 \, \phi _{0}^{-0.67}$ for the range $\phi _{0} \in [8\times 10^{-2},10^{3}]$, see the dashed lines in Fig. 12a. The transition between the power law relations occurs over the range $10^{-4} \le \phi _{0} \le 10^{0}$. For a given value of $\phi _{0}$, $C_{s}$ lies between the two limiting values. The power-law fit for high values of $\phi _{0}$ is slightly shallower than that for the stiff limit described above, indicating that response tends to this limit for values of $\phi _{0}$ in excess of $10^{6}$. In this limit the rate of deformation in the damage zone becomes very small compared to that in the surrounding matrix, which determines the stress distribution ahead of the crack tip and therefore the rate of growth of damage. There is no evidence of the data merging to the limiting result for low values of $\phi _{0}$, but the values of $\phi _{0}$ required to reach this limit are much lower than values we would expect from physical arguments (in this limit the material length scale is significantly greater that the geometric length scale—in practice we would expect any characteristic material length scale to be less than the geometric length scale for the cracked body, i.e. we would expect $\phi _{0}$ to be greater than 1). The power-law range for $\phi _{0}$ greater than $8\times 10^{-2}$ is therefore more representative of the physical behaviour of engineering components, so we concentrate on the relation for this regime here. Substituting this relationship into Eq. (30) gives the dimensionless velocity

52 $$\begin{aligned} \overset{\triangledown }{a} = 1.18 \times 10^{-2} \cdot \frac{\phi _{0}^{-0.77}}{\bar{\delta }_{n}^{\mathrm {f}}}. \end{aligned}$$

or into Eq. (31), the velocity in terms of $C^*$:

53 $$\begin{aligned} \dot{a}&= 9.0 \times 10^{-2} \cdot \frac{A^{0.77} \, \lambda ^{0.33}}{\delta _{n}^{\mathrm {f}} \, B^{0.67}} \, \left[ 0.56 \, C^* \right] ^{0.9} \nonumber \\&= 1.19 \times 10^{-2} \cdot \frac{A^{1.32} \, B^{0.23} \, \lambda ^{1.23} \, \sigma _{0}^{9}}{\delta _{n}^{\mathrm {f}}}. \end{aligned}$$

where we have substituted for $C^*$ using Eq. (21) to provide a relationship in terms of the reference stress $\sigma _{0}$. Figure 12a also shows a series of lines below which elastic effects can be ignored for $\sigma _{0}/E = 8 \times 10^{-6}$ (as used in the computations) and different values of critical crack tip opening displacement, i.e. below which inequality (51) is satisfied. As noted earlier this relationship is only valid for large values of $\phi _{0}$ (say greater than $8 \times 10^{-2}$). In this regime the computational results lie below this series of lines, indicating that the theoretical structure presented in Sect. 4 provides a valid framework for modelling the crack growth behavior.

We can repeat the analysis for plane strain conditions, see Fig. 12b. In the case of plane strain we again find that the results can be fit using two power-law relationships: $C_{s} = 0.55 \, \phi _{0}^{-0.05}$ over the range of values $\phi _{0} \in [10^{-10},10^{-2}]$ and $C_{s} = 0.19 \, \phi _{0}^{-0.29}$ for the range $\phi _{0} \in [10^{-2},10^{4}]$, see the dashed lines in Fig. 12b. Further, the transition between the power law relations takes place in the range $10^{-4} \le \phi _{0} \le 10^{0}$. The latter relation gives a dimensionless crack growth rate of

54 $$\begin{aligned} \overset{\triangledown }{a} = 2.5 \times 10^{-2} \cdot \frac{\phi _{0}^{-0.39}}{\bar{\delta }_{n}^{\mathrm {f}}}. \end{aligned}$$

The comparison between the FE results and the physical limits is also shown in Fig. 12b, which are again bounded by the physical limits of Eqs. (40) and (49), with the results asymptoting to Eq. (40) at large values of $\phi _{0}$. The large limit $\phi _{0}$ gives a faster crack growth rate in plane strain than plane stress (i.e. $C_{s}$ is larger for a given value of $\phi _{0}$) due to the higher stress levels ahead of a plane strain crack. The difference in slope between this limit and the computational results is greater than that observed for plane stress, but the value of $\phi _{0}$ where the two curves meet is about two orders of magnitude higher. As for plane stress, the results lie in a regime where elastic effects can be ignored.

### The effect of damage model

In order to investigate the effect of the detailed form of the damage zone model on the crack growth response, $\hat{C}_{k}$ has been determined for each of the different damage zone models described in Sect. 2. The parameters employed for these models are $m = 9$, $\delta _{n}^{\mathrm {f}} = 0.02$ $\mathrm {mm}$, $\beta = 1.0$, $h_{0} = 0.02$ $\mathrm {mm}$, $f_{0} = 0.01$ and $f_{\mathrm {c}} = 0.5$. It should be noted that $\delta _{n}$ is kept constant for all models. Further, we choosed $g_{0} = 2.23$ and $\beta =1.0$ such that all models yield the same rupture time $t_{\mathrm {f}}$ under a prescribed constant stress. (In “Appendix B” we demonstrate that under a given stress and for prescribed values of $\dot{\delta }_{0}$ and $\delta _{n}^{\mathrm {f}}$ the time to failure is proportional to a dimensionless quantity $\tilde{I}_{k}$, see Eq. (B.6). We choose the values $\beta$ and $g_{0}$ in the exponential Kachanov and micromechanical models such that $\tilde{I}_{k}$, and therefore the time to failure, is the same for all the models). In the studies of crack growth, the physical length scale of the cracked body $\lambda = H/2$ is used and the matrix rate sensitivity parameter is taken to be $n = 9$, as before. Here, we limit our consideration to plane stress conditions, but similar results can be obtained under plane strain. $\hat{C}_{k}$ is determined using the same procedure as described above, by comparing the computed steady state crack growth rate with Eq. (34). As before, we can determine analytical relationships for the response in the limits of small and large $\phi _{0}$. In Sect. 4.3 we found that the analytical model in the limit as $\phi _{0}\rightarrow 0$ does not provide a meaningful bound to the results and we do therefore do not present results in this limit for the remaining damage zone models described in Sect. 2. The analytical results for these models in the limit $\phi _{0}\rightarrow \infty$ are presented in “Appendix C”.

**Fig. 13:**
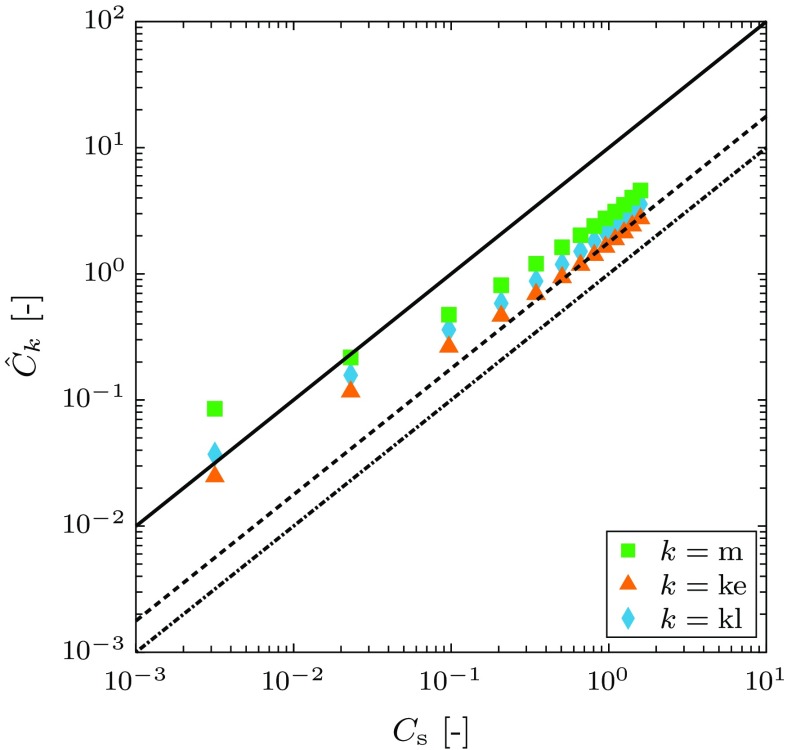
The relation between $\hat{C}_{k}$ and $C_{\mathrm {s}}$ (which is a function of $\phi _{0}$ parameter—see Fig. 12a for the different models under plane stress conditions. The *solid line* represent the response given by the model presented in “Appendix C”. The *dashed line* represent the apparent asymptotic behaviour. The *chained line* corresponding to $\mu _{k}=1$ represents a strict lower bound to the data

Rather than express $\hat{C}_{k}$ as a function of $\phi _{0}$ it proves instructive to express it as a function of $\hat{C}_{k}$ (which is a function of $\phi _{0}$) for each of the models. Figure 13 shows the relationship between $\hat{C}_{k}$ and $\hat{C}_{\mathrm {s}}$. The results show that these relations are nonlinear. However, for each model there is* point-wise* a linear relationship between these two functions with

55 $$\begin{aligned} \hat{C}_{k} = \mu _{k} \,\hat{C}_{\mathrm {s}}. \end{aligned}$$

where $\mu _{k}$ is a parameter that depends on the damage model used. The effect of damage is to soften the constitutive response, effectively increasing the effective separation rate across the damage zone for a given traction $T_{n}$, which results in a lower effective value of $\phi _{0}$ and therefore an increase of the crack growth rate. Therefore $\mu _{k}$ is larger than 1.0 for a given value of $\delta _{n}^{\mathrm {f}}$. Values $\mu _{k}$ are given in “Appendix C” for the three models: $\mu _{\mathrm {kl}}=\mu _{\mathrm {ke}} = \mu _{\mathrm {m}} = 10$. Substituting these values of $\mu _{k}$ into Eq. (55) give the straight lines plotted in Fig. 13, which also shows the computational results.

The computational results approach the analytical results for small values of $\hat{C}_{\mathrm {s}}$, i.e. large values of $\phi _{0}$, see Fig. 13, which corresponds to the limit where we would expect the analytical result to apply. As is increased gradually reduces for all the models and then asymptotes to a value $\mu _{\mathrm {kl}}=\mu _{\mathrm {ke}} = \mu _{\mathrm {m}} = 1.8$. As $\hat{C}_{\mathrm {s}}$ increases deformation in the damage zone becomes constrained and the stress relaxes more for a damaged material for a given value of $\phi _{0}$ compared to a material that does not damage. Therefore, the elevation of the crack growth rate is less. In the limit that deformation is completely constrained [corresponding to the situation considered by Cocks and Ashby (1981)] the crack velocity is independent of the details of the model and only depends on the critical opening displacement $\delta _{n}^{\mathrm {f}}$; thus in this limit we would expect $\mu _{k}$ to equal 1 for all the models considered here. This represents a physical lower bound to $\mu _{k}$, which is not reached for any of the models with the results appearing to asymptote to a higher limiting value over the range of conditions considered in the computations.

### Comparison with experimental data

**Fig. 14:**
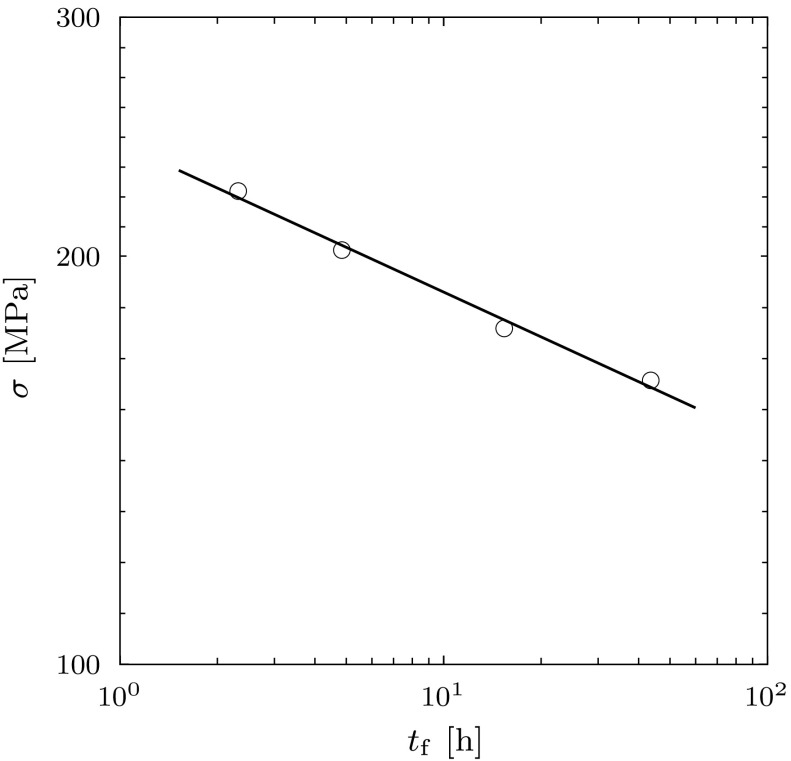
Comparison between uniaxial creep rupture data for 2.25 $\mathrm {Cr}$ $\mathrm {Mo}$ steel at 538 $^\circ \mathrm {C}$ and the interface damage model in (B.6). ‘$\bigcirc$’ represents the experimental data and the *black line* indicates the model predictions

**Fig. 15:**
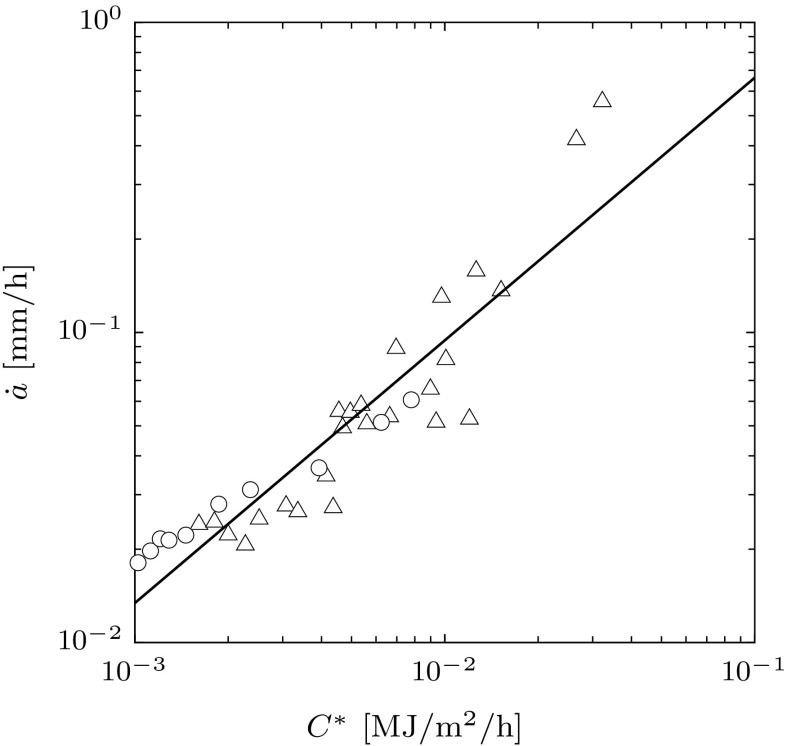
Comparison between experimental creep crack growth data for 2.25 $\mathrm {Cr}$ $\mathrm {Mo}$ steel at 538 $^\circ \mathrm {C}$ and the proposed framework. ‘$\bigcirc$’ and ‘$\bigtriangleup$’ represents the exprimental data for $B_{\mathrm {n}}=6$ $\mathrm {mm}$ and $B_{\mathrm {n}}=10$ $\mathrm {mm}$, respectively, and $B=25$ $\mathrm {mm}$. The *black line* indicates the framework predictions for the different damage models

The main objective of this paper is to identify simple constitutive models for the damaging process ahead of a crack tip in a creeping material and to identify a simple structural configuration which can be analysed rigorously to provide new insights into the relationship between damage models and the crack growth process, including the role of different characteristic material and geometric length scales. As a result, the simple geometry and loading conditions considered are not representative of laboratory test components. None-the-less, it proves instructive to explore how the models presented here can be calibrated against available experimental data, to determine the characteristic material and geometric length scales in these experiments and explore where this data lies with respect to the general trends identified in Figs. 14 and 15.

In this section, we consider the low alloy steel (2.25 $\mathrm {Cr}$ $\mathrm {Mo}$ steel at 538 $^\circ \mathrm {C}$) investigated by Nikbin et al. (1983). They provide data for creep deformation, creep rupture as well as creep crack growth generated using compact tension (CT) specimens, see Figs. 14 and 15. Consider the creep crack law of Eq. (31), together with the definition of $\phi _{0}$ in Eq. (32) or following Eq. (26). In order to determine the crack growth rate we need to determine the characteristic length $\lambda$ for the cracked geometry and the material parameters *n*, *m*, $\delta ^{\mathrm {f}}_{n}$ and $\dot{\varepsilon }_{0}$, $\dot{\delta }_{0}$ (at a reference stress $\sigma _{0}$) or equivalently the material parameters *A* and *B*. The steady creep response under a constant uniaxial stress $\sigma$ is given by Nikbin et al. (1983) at 538 $^\circ \mathrm {C}$:

56 $$\begin{aligned} \dot{\varepsilon } = \dot{\varepsilon }_{0} \, \left( \dfrac{\sigma }{\sigma _{0}} \right) ^n = B \, \sigma ^n, \end{aligned}$$

where $$n=9$$ and $$B=10^{-23}$$ $$\mathrm {MPa}^{-9} \, \mathrm {h}^{-1}$$.

In the remainder of the fitting process described in detail here we limit our consideration to the linear Kachanov model. Parallel procedures can be undertaken for the other damage models described in this paper. In determining the material parameters we assume that the damage zone model can also be used to describe damage development on grain boundaries in a uniaxial test. We further assume that damage grows primarily on boundaries normal to the direction of the applied stress. Integrating the damage growth rate equation between the limits $\omega = 0$ at time $t = 0$ and $\omega = 1$ at failure, i.e. when $t = t_{\mathrm {f}}$, then gives (see “Appendix B”)

57 $$\begin{aligned} t_{\mathrm {f}} \cdot \sigma ^{m} = \dfrac{\sigma _{0}^{m}}{m+1} \, \left( \dfrac{\delta ^{\mathrm {f}}_{n}}{\dot{\delta }_{0}}\right) = \dfrac{1}{m+1} \, \left( \dfrac{\delta ^{\mathrm {f}}_{n}}{A}\right) = D. \end{aligned}$$

Creep rupture data given by Nikbin et al. (1983) is plotted in Fig. 14, which gives $m = 9$, $D = 2.7 \times 10^{22}$ $\mathrm {MPa}^9\cdot \mathrm {h}$, thus providing a relationship between two of the material parameters

58 $$\begin{aligned} \delta ^{\mathrm {f}}_{n} = 2.7 \times 10^{23} \, A \, \left[ \mathrm {mm}\right] . \end{aligned}$$

where *A* is measured in units of $\mathrm {mm}/( \mathrm {MPa}^{9} \cdot \mathrm {h})$.

In the second step, we determine another relationship between $\delta ^{\mathrm {f}}_{n}$ and *A* from fitting the creep crack growth data ($\dot{a}$ vs $C^{*}$) to the model in Eq. (31). This combined with Eq. (58) provides two equations in terms of the two unknowns $\delta ^{\mathrm {f}}_{n}$ and *A*. To do this, we must represent Eq. (31) in terms of the fitting parameters $\delta ^{\mathrm {f}}_{n}$ and *A*. To do this we also need to determine the geometric length scale for the compact tension specimen employed in the crack growth studies. This requires the identification of an expression for $C^{*}$. Here we employ an expression employed in the UK R5 assessment procedure (Ainsworth et al. 1987), which is equivalent in form to the relationship derived for the double cantilever beam (Eq. 21), i.e.

59 $$\begin{aligned} C^{*} = \dot{\varepsilon }_{0} \, \sigma _{0} \, \lambda , \end{aligned}$$

where $\dot{\varepsilon }_{0}$ is the uniaxial strain rate at a reference stress $\sigma _{0}$ (Ainsworth et al. 1987). The reference stress is defined by

60 $$\begin{aligned} \sigma _{0} = \dfrac{P}{P_{\mathrm {L}}} \, \sigma _{\mathrm {y}}, \end{aligned}$$

where $P_{\mathrm {L}}$ is the limit load for a perfectly plastic material of yield strength $\sigma _{\mathrm {y}}$ and *P* is the applied load. The characteristic length scale $\lambda$ for a component is defined by $\lambda = K_{\mathrm {I}}^{2}/\sigma _{0}^{2}$ where $K_{\mathrm {I}}$ is the stress intensity factor for the specimen at the applied load *P*. For a compact tension specimen the limit load for the case of plane stress (Miller and Ainsworth 1989) is given by

61 $$\begin{aligned} P_{\mathrm {L}} = \sigma _{\mathrm {y}} \cdot W \cdot \underbrace{\left\{ \left[ \left( 1+\gamma \right) \cdot \left( 1+\gamma \left( \dfrac{a}{W}\right) ^{2}\right) \right] ^{\frac{1}{2}}- \left( 1+ \dfrac{a}{W} \right) \right\} }_{\varUpsilon _{\mathrm {L}}}, \end{aligned}$$

where $\varUpsilon _{\mathrm {L}}$ is a shape function, $\gamma = 1.155$, *a* is the crack length and *W* is the width of the specimen. The mode I stress intensity factor is given by

62 $$\begin{aligned} K_{\mathrm {I}} = \dfrac{P}{W^{\frac{1}{2}}} \cdot \varUpsilon _{\mathrm {K}}, \end{aligned}$$

where the shape function $\varUpsilon _{\mathrm {K}}$ is given by

63 $$\begin{aligned} \varUpsilon _{\mathrm {K}}&= \dfrac{2+\dfrac{a}{W}}{\left( 1-\dfrac{a}{W}\right) ^{\frac{3}{2}}} \cdot \left[ 0.886+4.64 \cdot \left( \dfrac{a}{W}\right) - 13.32 \cdot \left( \dfrac{a}{W}\right) ^{2} \right. \nonumber \\&\quad \left. +\,14.72 \cdot \left( \dfrac{a}{W}\right) ^{3} - 5.60 \cdot \left( \dfrac{a}{W}\right) ^{4} \right] . \end{aligned}$$

Hence, the characteristic length scale is given by

64 $$\begin{aligned} \dfrac{\lambda }{W} = \left( \varUpsilon _{\mathrm {L}} \cdot \varUpsilon _{\mathrm {K}}\right) ^{2}. \end{aligned}$$

Miller and Ainsworth (1989) compared the predictions of Eq. (59) with detailed finite element calculations and suggested a modification to this expression to provide a better agreement with the computational results:

65 $$\begin{aligned} C^{*} = \dot{\varepsilon }_{0} \, \sigma _{0} \, \lambda \, F_{\mathrm {p}}^{n+1}. \end{aligned}$$

where $F_{\mathrm {p}}$ is a dimensionless parameter which is in the range 0.92 to 0.96 for $n=9$ and *a* / *W* in the range 0.25 to 0.5. Here we use an average value of 0.94. Equation (65) effectively reduces the reference stress by a factor $F_{\mathrm {p}}$, but does not change the expression for the characteristic length.

**Table 1:** The damage models parameters

Interface model, *k*	*m* [−]	*A* [$\mathrm {mm} / (\mathrm {MPa}^{9} \, \mathrm {h})$]$^{\ddagger }$	$\delta _{n}^{\mathrm {f}}$ [$\mu \mathrm {m}$]$^{\dagger }$	$\beta$ [−]	$h_{0}$ [$\mu \mathrm {m}$]	$f_{0}$ [−]	$f_{\mathrm {c}}$ [−]
$\mathrm {kl}$	9	$8.84 \times 10^{-27}$	$1.10 \times 10^{2}$	–	–	–	–
$\mathrm {ke}$	9	$4.15 \times 10^{-25}$	$2.35 \times 10$	–	–	–	–
$\mathrm {m}$	9	$1.71 \times 10^{-26}$	$4.56 \times 10$	–	1.88	0.00032	0.71

${}^\dagger$ $\delta _{n}^{\mathrm {f}}$ is calculated using Eq. (13) for the micromechanical model

${}^\ddagger$ Note, $\sigma _{0}$ changes during the duration of a test, causing $\dot{\varepsilon }_{0}$ and $\dot{\delta }_{0}$ to also change. At a given instant $\sigma _{0}$ can be determined from Eq. (60), multiplied by $F_{\mathrm {p}}=0.94$, to take into account the correction of Miller and Ainsworth (1989)

For the CT specimen tested by Nikbin et al. (1983) $W=50$ $\mathrm {mm}$ and the initial crack length was $a_{0}=12.5$ $\mathrm {mm}$. The crack growth rate is plotted as a function of $C^{*}$ in Fig. 15, which covers a 2 orders of magnitude increase in $C^{*}$ over the period of stable crack growth. From Eq. (64) we find that a two orders of magnitude increase in $C^{*}$ corresponds to an increase of crack length from *a* / *W* to 0.25 to 0.4. From Eq. (64) we find that this corresponds to a change of characteristic length from $\lambda /W = 1.3$ to 1.0. In our evaluation of the data we take an average of these values for the characteristic length, i.e. $\lambda /W = 1.15$. In order to proceed we need to determine which expressions to use for $\hat{C}_{k}$ in Eq. (31), i.e. which regions of Figs. 12a and 13 the data lies in. From the creep deformation and creep rupture data presented earlier we find $\dot{\varepsilon }_{0} = 1.24 \times 10^{-24} \, \sigma _{0}^{9}$ $1/\mathrm {h}$ and $\phi _{0} = 2.17 \times 10^{2}/\delta _{n}^{\mathrm {f}}$ [defined after Eq. (26)], which suggests that for typical expected critical opening displacements in metals ($\delta _{n}^{\mathrm {f}} \in [10^{-10}-10^{-5}]$ $\mathrm {m}$, i.e. of the order of the mean cavity spacing) $\phi _{0} > 1.0$. Therefore, we use the power-law relation for $\phi _{0} > 1.0$ which is $\hat{C}_{\mathrm {kl}} = 0.40 \cdot \phi _{0}^{-0.494}$. In this regime, Eq. (31) can be written in the form

66 $$\begin{aligned} \dot{a} = 1.18 \times 10^{-5} \cdot \dfrac{{C^{*}}^{0.9}}{{\delta _{n}^{\mathrm {f}}}^{0.41}}, \end{aligned}$$

where $\dot{a}$ is in $\mathrm {mm/h}$, $\delta _{n}^{\mathrm {f}}$ is in $\mathrm {mm}$ and $C^{*}$ is in $\mathrm {MJ/mm2/h}$. Fitting this expression to the data of Fig. 15 gives the critical separation $\delta _{n}^{\mathrm {f}}$. The rate parameter *B* can then be determined from Eq. (58). We can employ the same fitting procedure for the exponential Kachanov and micromechanical models. In these models additional parameters are required, i.e. $\beta$ for the exponential Kachanov model and $h_{0}$, $f_{0}$ and $f_{\mathrm {c}}$ for the micromechanical model. For $\phi _{0} > 1.0$, $\hat{C}_{\mathrm {ke}} = 0.30 \cdot \phi _{0}^{-0.515}$ and $\hat{C}_{\mathrm {m}} = 0.49 \cdot \phi _{0}^{-0.373}$ for the exponential Kachanov and micromechanical models. Using a nonlinear least squares method, the crack growth rate in Eq. (66) is then fitted to the creep crack growth data. Figure 15 shows the comparison between experimental creep crack growth data and the framework predictions for different damage models (they all lie on the same straight line and yield comparable goodness of fit). The fitting parameters are shown in Table 1 for the different models. The parameters indicate that the material data falls in regime close to the stiff limit, i.e. $\phi _{0} \in [10^{3}-10^{4}]$. Further, the failure separation falls within the physical regime, i.e.

In the above analysis we noted that the characteristic length scale only changes by a small amount during the course of an experiment (i.e. by less than 13% from the mean value), while $C^{*}$ changes by over 2 orders of magnitude, thus the effect of $C^{*}$ swamps that due to $\lambda$ and we could assume a constant value of $\lambda$ when fitting the data. We need to be careful, however when using the developed model to assess the growth of defects in high temperature components. In general, any defects of interest will be much smaller than that employed in laboratory experiments, such as the CT specimens considered here. Conventional models of creep crack growth determined from experimental data do not include the effect of $\lambda$ on the crack growth rate, other than how it affects $C^{*}$ (i.e. experimental laws generally assume that $\dot{a} \propto {C^{*}}^{q}$, where $q < 1$). This has two major consequences. Firstly, the models developed from the data give a crack growth rate that is proportional to $\lambda ^{p}$, where *p* is of the order of 0.5. Thus as the crack size is reduced the crack growth rate reduces compared with that determined from the laboratory data for the same value of $C^{*}$. Thus use of laboratory data overestimates the crack growth rate. Secondly, as $\lambda$ is reduced $\phi _{0}$ also reduces and damage growth ahead of a crack tip becomes more constrained, with the loading condition moving to the left on Fig. 12 and towards the top right corner on Fig. 13. This can result in a transition to the low $\phi _{0}$ regime where now $p \approx 1.0$. The crack growth rate is now more sensitive to the size of the defect and the laboratory data provides an even more significant overestimation of the crack growth rate.

## Concluding remarks

In this study, a combined theoretical and computational study for crack growth under steady state conditions is presented. A theoretical framework is introduced in which the constitutive behaviour of the bulk material is described by power-law creep. A new class of damage zone models is proposed to model the fracture process such that the constitutive relation is described by a traction-separation rate law. Further, three different damage zone models are investigated: a simple critical displacement model; empirical Kachanov damage type models; and a micromechanical model. The path independence of the $C^{*}$-integral is used to relate the far field loading to the damage growth process along a narrow zone directly ahead of the growing crack tip. A double cantilever beam specimen (DCB) subjected to constant pure bending moment is studied and analytical models are developed for pure mode-I steady-state crack growth. Further, using dimensional analysis the dimensionless functions $C_{\mathrm {s}}$ and $\hat{C}_{k}$ are derived which account for the detailed form of the model on the crack growth process. A computational framework is then implemented using the Finite Element method and the analytical models are calibrated against detailed Finite Element simulations, allowing the $C_{\mathrm {s}}$ and $\hat{C}_{k}$ functions to be determined for different combinations of dimensionless parameters. The $\hat{C}_{k}$ function depends on the ratio of geometric to material length scales for the problem, $\phi _{0}$, and the rate sensitivity exponents *n* and *m*. The $C_{\mathrm {s}}$-function is found to take two different power law forms over a wide range of values of $\phi _{0}$. Two simple models are presented by consider the response as $\phi _{0}\rightarrow 0$ and $\phi _{0}\rightarrow \infty$, which bound the computational results. The crack velocity asymptotes to the $\phi _{0}\rightarrow \infty$ limit as $\phi _{0}$ increases. Incorporation of the effect of damage into the constitutive response for the damage zone, results in a more substantial relaxation of stress ahead of the crack tip and a faster crack growth rate for a given value of $\phi _{0}$ and a delay in the asymptote to the $\phi _{0}\rightarrow \infty$ limit. Comparison with experimental data for a 2.25 $\mathrm {Cr}$ $\mathrm {Mo}$ steel at 538 $^\circ \mathrm {C}$ shows that the different damage models are capable of fitting creep crack growth data using physically reasonable parameters.

In this paper we have used simple material models for the matrix and damage zone response to analyses crack growth in a simple geometry. Nonetheless, the calculations provide a useful framework for evaluating the effect of different damaging processes on crack growth and evaluating data for more complex laboratory specimens.

## Figures and Tables

**Fig. 16:**
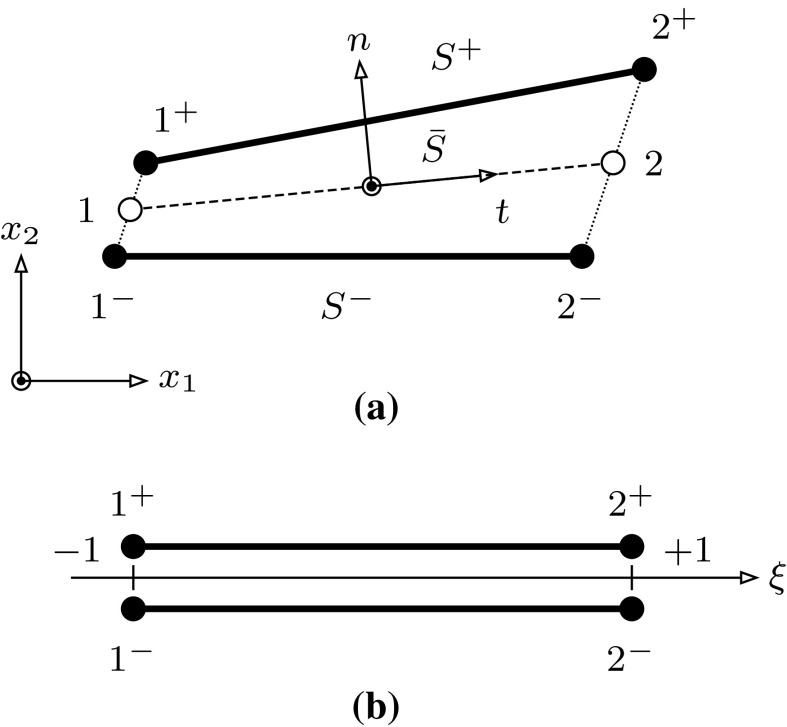
Geometry of an interface element: **a** the physical domain and **b** the parent domain. The definition of the surfaces $S^{-}$, $S^{+}$ and $\bar{S}$ that coincide in the reference configuration

## Appendix: Finite Element formulation of the interface element

The interface damage models in Sect. 2.3 have been implemented into the Finite Element method using cohesive element approach. For the implementation purposes, the traction-separation rate law is assumed to be determined by elastic and creep responses, i.e. the total separation is decomposed into elastic and creep contributions. Therefore, the total separation rates are written as

A.1 $$\begin{aligned} \dot{\delta }_{i} = \dot{\delta }^{\mathrm {el}}_{i}+\dot{\delta }^{\mathrm {cr}}_{i} \end{aligned}$$

where $i \in \left[ t, n\right]$ and $\dot{\delta }^{\mathrm {el}}_{i}$ and $\dot{\delta }^{\mathrm {cr}}_{i}$ are the elastic and creep separation rates, respectively. Additionally, this study is limited to pure mode I loading, the creep behaviour is only considered in the normal direction and the response of the tangential direction is taken to be purely elastic. The traction-separation rate laws for the tangential and normal directions are respectively defined as

A.2 $$\begin{aligned} \dot{\delta }_{i} = \dot{\delta }^{\mathrm {el}}_{n} = \dfrac{\dot{T_{t}}}{K_{t}}, \end{aligned}$$

and

A.3 $$\begin{aligned} \dot{\delta }_{n} = \dot{\delta }^{\mathrm {el}}_{n}+\dot{\delta }^{\mathrm {cr}}_{n} = \dfrac{\dot{T_{n}}}{K_{n}} + \dot{\delta }_{0} \, \left( \dfrac{T_{n}}{d_{k} \, T_{0}} \right) ^{m}. \end{aligned}$$

where $K_{t}$ and $K_{n}$ are the elastic stiffness parameters in the tangential and normal directions, respectively. The damage function $d_{k}$ depends on the normal creep separation $\delta ^{\mathrm {cr}}_{n}$.

The surface-like cohesive formulation is adopted in this work (e.g. see Willam et al. 1989; Ortiz and Suresh 1993; Xu and Needleman 1993; Ortiz and Pandolfi 1999). In this formulation, a cohesive element consists of two surface elements which coincide in the reference configuration. The upper and lower surface elements are denoted $S^{+}$ and $S^{-}$, respectively, and the constitutive behaviour of the cohesive element is defined in a middle surface $\bar{S}$. A 4-node two-dimensional linear element is used in this study. The middle surface is defined by the two points 1–2 which are the mid-points between nodes $1^{-}$–$2^{-}$ and $1^{+}$–$2^{+}$ in the lower and upper elements, respectively, see Fig. 16. Hence, the middle surface is then defined by the interpolation

A.4 $$\begin{aligned} \text{ x }= \sum \limits _{a=1}^{2} N_{a} \, \bar{\text{ x }}_{a}, \end{aligned}$$

where $\bar{\text{ x }}_{a}$ is the current coordinates vector of the middle surface which is related to the current nodal coordinates of the upper and lower surface elements $\text{ x }^{+}_{a}$ and $\text{ x }^{-}_{a}$, respectively, by

A.5 $$\begin{aligned} \bar{\text{ x }}_{a} = \frac{1}{2} \left( \text{ x }^{+}_{a} + \text{ x }^{-}_{a} \right) . \end{aligned}$$

The standard shape functions, $N_{a} = N_{a}^{-} = N_{a}^{+}$, are defined in the local coordinates, $\xi \in \left[ -1, 1\right]$, as

A.6 $$\begin{aligned} N_{1}&= \frac{1}{2} \left( 1-\xi \right) , \end{aligned}$$

A.7 $$\begin{aligned} N_{2}&= \frac{1}{2} \left( 1+\xi \right) . \end{aligned}$$

Similarly, the global displacement jump across the cohesive element, $\bar{\text{ u }}$, is defined in points 1 and 2 as

A.8 $$\begin{aligned} \text{ u }= \sum \limits _{a=1}^{2} N_{a} \, \bar{\text{ u }}_{a}, \end{aligned}$$

where $\bar{\text{ u }}_{a}$ is the displacement jumps of the middle surface which is related to the nodal displacement by

A.9 $$\begin{aligned} \bar{\text{ u }}_{a} = \text{ u }^{+}_{a} - \text{ u }^{-}_{a}. \end{aligned}$$

The finite element formulation is developed from principle of virtual work where the nodal forces and the stiffness matrix are computed. Hence, the principle of virtual work for the interface zone is defined as

A.10 $$\begin{aligned} \delta \Pi _{\mathrm {int}} = \int \limits _{\varGamma _{\mathrm {int}}} \text{ T }\, \delta \varvec{\delta }\cdot \mathrm {d}S, \end{aligned}$$

where $\varGamma _{\mathrm {int}}$ is the interface surface, $\text{ T }$ is the traction vector across the interface and $\delta \varvec{\delta }$ is the virtual separation that are defined in the local coordinate system. The internal nodal force across the interface surface is determined from the tractions and by substitution of the finite element interpolation into Eq. (A.10) as

A.11 $$\begin{aligned} \text{ f }_{\mathrm {int}}^{\pm a} = \int \limits _{\varGamma _{\mathrm {int}}} \text{ T }\, N_{a} \cdot \mathrm {d}S. \end{aligned}$$

The consistent tangent stiffness matrix is determined from the linearization of Eq. (A.11), with the result

A.12 $$\begin{aligned} \text{ K }_{\mathrm {int}}^{\pm } = \dfrac{\partial \text{ f }_{\mathrm {int}}^{\pm a}}{\partial \varvec{\delta }_{b}} = \int \limits _{\varGamma _{\mathrm {int}}} N_{a} \, \dfrac{\partial \text{ T }}{\partial \varvec{\delta }} \, N_{b} \cdot \mathrm {d}S. \end{aligned}$$

It should be noted that the nodal forces and the consistent tangent matrix are defined in the local coordinate system (*t*, *n*) and a transformation to the global system (1, 2) is needed before assembling to the finite element equations. This transformation includes mapping from the local to the global coordinates and relating the local to the global nodal numbering.

### The cohesive element traction

The tractions are determined using the incremental forms of the total separation rates in Eqs. (A.2) and (A.3). Hence, a set of equations is obtained from these incremental form at the $i+1$th increment as

A.13 $$\begin{aligned} H_{t}&= \varDelta \delta _{t} - \dfrac{\varDelta T_{t}}{K_{t}} = 0, \end{aligned}$$

A.14 $$\begin{aligned} H_{n}&= \varDelta \delta _{n} - \dfrac{\varDelta T_{n}}{K_{n}} + \varDelta t \, \dot{\delta }_{0} \, \left( \dfrac{T_{n}^{i}+\varTheta \varDelta T_{n}}{d_{k} \, T_{0}} \right) ^{m} = 0, \end{aligned}$$

where $\varDelta t$ is the time increment, $T_{i}^{n}$ is the normal traction at the start of the increment and $\varTheta$ is integration parameter ($1 \ge \varTheta \ge 0$).

In order to solve these equations, we use encapsulate the equations in a vector $\text{ H }= \left[ H_{t} \quad H_{n}\right] ^{\mathrm {T}}$ and define the unknown vector $\varDelta \text{ T }= \left[ \varDelta T_{t} \quad \varDelta T_{n}\right] ^{\mathrm {T}}$. Using Newton-Raphson algorithm, the following linearized form of these equations is iteratively solved for the traction increment until a convergence condition is achieved

A.15 $$\begin{aligned} \text{ H }\left( \varDelta \text{ T }+ \delta \varDelta \text{ T }\right) = \text{ H }\left( \varDelta \text{ T }\right) + \underbrace{\dfrac{\partial \text{ H }}{\partial \varDelta \text{ T }}}_{\text{ J }} \, \delta \varDelta \text{ T }= \text{0 } \end{aligned}$$

where $\text{0 }$ is a zeros vector ($2 \times 1$) and the Jacobian $\text{ J }$ is defined as

A.16 $$\begin{aligned} \text{ J }= \left[ \begin{array}{ll} \dfrac{\partial H_{t}}{\partial \varDelta T_{t}} &{} \dfrac{\partial H_{t}}{\partial \varDelta T_{t}} \\ \dfrac{\partial H_{n}}{\partial \varDelta T_{t}} &{} \dfrac{\partial H_{n}}{\partial \varDelta T_{n}} \end{array}\right] , \end{aligned}$$

where

A.17 $$\begin{aligned} \dfrac{\partial H_{t}}{\partial \varDelta T_{t}}&= - \dfrac{1}{K_{t}}, \end{aligned}$$

A.18 $$\begin{aligned} \dfrac{\partial H_{t}}{\partial \varDelta T_{n}}&= 0, \end{aligned}$$

A.19 $$\begin{aligned} \dfrac{\partial H_{n}}{\partial \varDelta T_{t}}&= 0, \end{aligned}$$

A.20 $$\begin{aligned} \dfrac{\partial H_{n}}{\partial \varDelta T_{n}}&= - \dfrac{1}{K_{n}}- \varDelta t \, \dfrac{\dot{\delta }_{0}}{d_{k} \, T_{0}} \, \varTheta \, \left( m-1\right) \nonumber \\&\qquad \times \left[ \dfrac{T_{n}^{i}+\varTheta \varDelta T_{n}}{d_{k} \, T_{0}} \right] ^{m-1}. \end{aligned}$$

### The cohesive zone Jacobian

The definition of the cohesive zone Jacobian in Eq. (A.12) yields interface tangent modulus as

A.21 $$\begin{aligned} \mathbb {C} = \dfrac{\partial \varDelta \text{ T }}{\partial \varDelta \varvec{\delta }} = \left[ \begin{array}{c c} \dfrac{\partial \varDelta T_{t}}{\partial \varDelta \delta _{t}} &{} \dfrac{\partial \varDelta T_{t}}{\partial \varDelta \delta _{t}} \\ \dfrac{\partial \varDelta T_{n}}{\partial \varDelta \delta _{t}} &{} \dfrac{\partial \varDelta T_{n}}{\partial \varDelta \delta _{n}} \end{array}\right] , \end{aligned}$$

The elements of the tangent modulus can be determined from the variations of the incremental forms in Eqs. (A.14). The variation of $H_{i}$ is given by

A.22 $$\begin{aligned} \delta H_{i} = \dfrac{\partial H_{i}}{\partial \varDelta \delta _{t}} \cdot \delta \varDelta \delta _{t} + \dfrac{\partial H_{i}}{\partial \varDelta \delta _{n}} \cdot \delta \varDelta \delta _{n} = 0 \end{aligned}$$

where the variations of the separation increments $\delta \varDelta \delta _{t}$ and $\delta \varDelta \delta _{n}$ can be chosen arbitrarily yielding that $\partial H_{i}/\partial \varDelta \delta _{t} = 0$ and $\partial H_{i}/\partial \varDelta \delta _{n} = 0$. Hence, the variations of Eqs. (A.14) gives

A.23 $$\begin{aligned} \dfrac{\partial H_{t}}{\partial \varDelta T_{t}}&= \dfrac{1}{K_{t}}, \end{aligned}$$

A.24 $$\begin{aligned} \dfrac{\partial H_{t}}{\partial \varDelta T_{n}}&= 0, \end{aligned}$$

A.25 $$\begin{aligned} \dfrac{\partial H_{n}}{\partial \varDelta T_{t}}&= 0, \end{aligned}$$

A.26 $$\begin{aligned} \dfrac{\partial H_{n}}{\partial \varDelta T_{n}}&= \dfrac{1+\varDelta t \, \dfrac{\dot{\delta }_{0}}{d_{k} \, T_{0}} \, \varTheta \, \left( m-1\right) \, \left[ \dfrac{T_{n}^{i}+\varTheta \varDelta T_{n}}{d_{k} \, T_{0}} \right] ^{m} \dfrac{\partial d_{k}}{\partial \varDelta \delta _{n}}}{\dfrac{1}{K_{n}}+\varDelta t \, \dfrac{\dot{\delta }_{0}}{d_{k} \, T_{0}} \, \varTheta \, \left( m-1\right) \, \left[ \dfrac{T_{n}^{i}+\varTheta \varDelta T_{n}}{d_{k} \, T_{0}} \right] ^{m-1}}. \end{aligned}$$

In the case of high values of $K_{n}$ the derivative of the damage function for good approximation can be taken as $\partial d_{k} / \partial \varDelta \delta _{n} \approx \partial d_{k}/ \partial \varDelta \delta _{n}^{\mathrm {cr}}$.

## Appendix: Prediction of interface damage

In this appendix we will develop relations for interface damage. Consider a constant uniaxial stress state given by $T_{n}= \sigma$ such that damage is accumlated from an initial value to complete failure in a time $t_{\mathrm {f}}$ (rupture life), i.e. $\omega \in [0,1]$ and $f \in [f_{0},f_{\mathrm {c}}]$. The rupture life is obtained by integration of the relation between the damage parameter and the traction which is derived from the relationship between $\dot{\delta }_{n}$ and $T_{n}$ and the damage evolution law. A general form of the relationship between $\dot{\delta }_{n}$ and $T_{n}$ is expressed as

B.1 $$\begin{aligned} \dot{\delta }_{n} = \dot{\delta }_{0} \left( \dfrac{T_{n}}{d_{k} \, T_{0}}\right) ^{m}, \end{aligned}$$

where the function $d_{k}$ is given in Eq. (23) for the different interface models. Rearranging and integrating (B.1) gives

B.2 $$\begin{aligned} \int \limits _{\delta _{n}^{\mathrm {c}}}^{\delta _{n}^{\mathrm {f}}} {d_{k}}^{m} \cdot \mathrm {d}\delta _{n} = \dot{\delta }_{0} \left( \dfrac{\sigma _{n}}{\sigma _{0}}\right) ^{m} \cdot \int \limits _{0}^{t_{\mathrm {f}}} \mathrm {d}t. \end{aligned}$$

where $\delta _{n}^{\mathrm {c}}$ is the opening at the initiation of damage and $\delta _{n}^{\mathrm {f}}$ is the critical opening. We assume $\delta _{n}^{\mathrm {c}} = 0$ for simplicity. The left-hand side of this equation, $I_{k} = \int {d_{k}}^{m} \cdot \mathrm {d}\delta _{n}$, is evaluated by replacing the separation increment $\mathrm {d}\delta _{n}$ with the damage parameter increment (i.e. $\mathrm {d}\omega$ or $\mathrm {d}f$) using the evolution laws in Eqs. (11), (12) and (15). Thus, for the case of Kachanov damage type models

B.3 $$\begin{aligned} I_{\mathrm {kl}} = \int \limits _{0}^{\delta _{n}^{\mathrm {f}}} {d_{\mathrm {kl}}}^{m} \cdot \mathrm {d}\delta _{n} = \int \limits _{0}^{1} \left( 1-\omega \right) ^{m} \, \delta _{n}^{\mathrm {f}} \cdot \mathrm {d}\omega =\dfrac{\delta _{n}^{\mathrm {f}}}{m+1}, \end{aligned}$$

and

B.4 $$\begin{aligned} I_{\mathrm {kl}} = \int \limits _{0}^{\delta _{n}^{\mathrm {f}}} {d_{\mathrm {ke}}}^{m} \cdot \mathrm {d}\delta _{n} = \int \limits _{0}^{1} \left( 1-\omega \right) ^{m} \, \dfrac{\delta _{n}^{\mathrm {f}}}{\beta } \cdot \mathrm {d}\omega =\dfrac{\delta _{n}^{\mathrm {f}}}{\beta \left( m+1\right) }, \end{aligned}$$

for the exponential damage model. In the case of micromechanical model the left-hand side reads

B.5 $$\begin{aligned} I_{\mathrm {m}}&= \int \limits _{0}^{\delta _{n}^{\mathrm {f}}} {d_{\mathrm {m}}}^{m} \cdot \mathrm {d}\delta _{n} = \dfrac{ \left( 1-f_{\mathrm {c}}\right) \, \left( 1-f_{0}\right) }{\left( f_{\mathrm {c}} -f_{0}\right) } \cdot \dfrac{\delta _{n}^{\mathrm {f}}}{g_{0}^{n+1}} \cdot \nonumber \\&\quad \int \limits _{f_{0}}^{f_{\mathrm {c}}} \dfrac{1}{\left( 1-f \right) ^{2}} \, \left[ \left( 1-f\right) ^2+\left( \frac{1}{\sqrt{3}} \, \ln \dfrac{1}{f}\right) ^2 \right] ^\frac{m+1}{2} \cdot \mathrm {d}f, \end{aligned}$$

where $I_{\mathrm {m}}$ is evaluated numerically. Thus, the stress-rupture relation becomes

B.6 $$\begin{aligned} \tilde{I}_{k} \cdot \dfrac{\delta _{n}^{\mathrm {f}}}{\dot{\delta }_{0}} = \left( \dfrac{\sigma }{\sigma _{0}}\right) ^{m} \cdot t_{\mathrm {f}}, \end{aligned}$$

where $$\tilde{I}_{k}=I_{k}/\delta _{n}^{\mathrm {f}}$$.

## Appendix: The damage effects on the physical limits

In this appendix we will develop relations for $\hat{C}_{k}$-function in the stiff physical limit in the presence of damage, i.e. similar to Eq. (40) in Sect. 4.3. The crack propagation rate is obtained by integration of the relation between the damage parameter and the traction which is derived from the relationship between $\dot{\delta }_{n}$ and $T_{n}$ and the damage evolution law as in “Appendix B”. Hence, recall the general form of relationship between $\dot{\delta }_{n}$ and $T_{n}$ in Eq. (B.1) and following the analysis presented in the main text we can replace (B.1) by

C.1 $$\begin{aligned} \int \limits _{\delta _{n}^{\mathrm {c}}}^{\delta _{n}^{\mathrm {f}}} {d_{k}}^{m} \cdot \mathrm {d}\delta _{n} = \left( n+1\right) \, \dfrac{\dot{\delta }_{0}}{\dot{a}} \, \left[ \dfrac{C^{*}}{\dot{\varepsilon }_{0} \, \sigma _{0} \, I_{n} \, r }\right] ^{\frac{n}{n+1}} \, \tilde{\sigma }_{\theta }\left( n,0\right) ^{n}, \end{aligned}$$

As before, we limit consideration to the case of $n=m$ and we assume $\delta _{n}^{\mathrm {c}} = 0$ for simplicity. The left-hand side is evaluated as in Eqs. (B.3), (B.4) and (B.5). The dimensionless velocity is determined as

C.2 $$\begin{aligned} \overset{\triangledown }{a} = \left( n+1 \right) \dfrac{\bar{\delta }_{n}^{\mathrm {f}} \, \bar{r}_{\mathrm {c}}^{\frac{1}{n+1}}}{\phi _{0} \, \bar{I}_{k}} \, \left[ \dfrac{f_{n}\left( n \right) }{I_{n}}\right] ^{\frac{n}{n+1}} \, \tilde{\sigma }_{\theta }\left( n,0\right) ^{n}, \end{aligned}$$

where $\bar{I}_{k}=I_{k}/\lambda$. Comparison with Eq. (30) yields the $\hat{C}_{k}$-function for the case of stiff interface as

C.3 $$\begin{aligned} \hat{C}_{k} = \left( n+1 \right) \, \dfrac{\bar{\delta }_{n}^{\mathrm {f}} \, \bar{r}_{\mathrm {c}}^{\frac{1}{n+1}}}{\bar{I}_{k}} \, \left[ \dfrac{2n}{n+1} \cdot \dfrac{1}{\phi _{0} \, I_{n}}\right] ^{\frac{n}{n+1}} \, \tilde{\sigma }_{\theta }\left( n,0\right) ^{n}. \end{aligned}$$

Comparison with $\hat{C}_{k}$-function in Eq. (34) and assuming that the size of the damage is similar:

C.4 $$\begin{aligned} \mu _{k} = \dfrac{\bar{\delta }_{n}^{\mathrm {f}}}{\bar{I}_{k}}. \end{aligned}$$

## Nomenclature

$2 \, l$: The spacing between two adjacent pores
$\beta$: A material parameter of the exponential damage law
$\delta ^{\mathrm {c}}_{n}$: The critical normal displacement jump in the damage zone at the crack tip
$\delta ^{\mathrm {f}}_{n}$: The normal displacement jump at failure in the crack tip
$\delta _i$: The displacement jump vector across the damage zone ($i=1,2,3$)
$\dot{\delta _{0}}$: The separation rate at the reference traction $T_{0}$
$\dot{\delta }^{\mathrm {m}}_{n}$: The maximum normal displacement jump rate vector in the crack tip
$\dot{\delta }_i$: The displacement jump rate vector across the damage zone ($i=1,2,3$)
$\dot{\varepsilon }_0$: The strain-rate at the reference stress $\sigma _0$
$\dot{a}$: The steady state crack velocity
$\hat{C}_{k}$: The separation history function of model *k*
$\lambda$: The characteristic geometric length scale
$\left( \bullet \right) ^{\mathrm {cr}}$: The creep component of the quantity $\left( \bullet \right)$
$\left( \bullet \right) ^{\mathrm {el}}$: The elastic component of the quantity $\left( \bullet \right)$
$\omega$: A scalar damage parameter
$\overset{\triangledown }{a}$: The dimensionless steady state crack velocity
$\phi _{0}$: The ratio of geometric to material length scales
$\sigma _0$: The reference stress
$\sigma _{\mathrm {e}}$: The von Mises equivalent stress
$\sigma _{ij}$: The Cauchy stress tensor
$\varepsilon _{ij}$: The engineering strain tensor
*a*: The crack length
$C^*$: The rate of the *J*-integral
$C_{s}$: The separation history function of the simple model
*E*: Young’s modulus
*f*: The current area fraction of the pores
$f_0$: The initial area fraction of the pores
$f_{\mathrm {c}}$: The coalescence area fraction of the pores
*h*: The current height of a pore
$h_0$: The initial height of the pores
*m*: The rate sensitivity exponent of the damage zone
*n*: The rate sensitivity parameter of the bulk material
$n_{i}$: The unit normal vector ($i=1,2,3$)
$s_{ij}$: The deviatoric part of Cauchy stress tensor
$T_i$: The traction vector ($i=1,2,3$)
$T_{0}$: The reference traction of the damage zone
$u_{i}$: The displacement vector ($i=1,2,3$)
$x_i$: The Cartesian material and spatial coordinates ($i=1,2,3$)
